# Dose Response, Dosimetric, and Metabolic Evaluations of Replacement PFAS Perfluoro-(2,5,8-trimethyl-3,6,9-trioxadodecanoic) Acid (HFPO-TeA)

**DOI:** 10.3390/toxics11120951

**Published:** 2023-11-22

**Authors:** Aero Renyer, Krishna Ravindra, Barbara A. Wetmore, Jermaine L. Ford, Michael DeVito, Michael F. Hughes, Leah C. Wehmas, Denise K. MacMillan

**Affiliations:** 1 Oak Ridge Institute for Science and Education (ORISE), Oak Ridge, TN 37830, USA; renyer.aero@epa.gov; 2 Oak Ridge Associated Universities (ORAU), Oak Ridge, TN 37830, USA; ravindra.krishna@epa.gov; 3 Center for Computational Toxicology and Exposure, Office of Research and Development, U.S. Environmental Protection Agency (EPA), Durham, NC 27709, USA; wetmore.barbara@epa.gov (B.A.W.); ford.jermaine@epa.gov (J.L.F.); devito.michael@epa.gov (M.D.); hughes.michaelf@epa.gov (M.F.H.); wehmas.leah@epa.gov (L.C.W.)

**Keywords:** PFAS, PFECA, thyroid disruption, dosimetry, non-targeted analysis (NTA), plasma protein binding, hepatic clearance, IVIVE

## Abstract

Few studies are available on the environmental and toxicological effects of perfluoroalkyl ether carboxylic acids (PFECAs), such as GenX, which are replacing legacy PFAS in manufacturing processes. To collect initial data on the toxicity and toxicokinetics of a longer-chain PFECA, male and female Sprague Dawley rats were exposed to perfluoro-(2,5,8-trimethyl-3,6,9-trioxadodecanoic) acid (HFPO-TeA) by oral gavage for five days over multiple dose levels (0.3–335.2 mg/kg/day). Clinically, we observed mortality at doses >17 mg/kg/day and body weight changes at doses ≤17 mg/kg/day. For the 17 mg/kg/day dose level, T3 and T4 thyroid hormone concentrations were significantly decreased (*p* < 0.05) from controls and HFPO-TeA plasma concentrations were significantly different between sexes. Non-targeted analysis of plasma and in vitro hepatocyte assay extractions revealed the presence of another GenX oligomer, perfluoro-(2,5-dimethyl-3,6-dioxanonanoic) acid (HFPO-TA). In vitro to in vivo extrapolation (IVIVE) parameterized with in vitro toxicokinetic data predicted steady-state blood concentrations that were within seven-fold of those observed in the in vivo study, demonstrating reasonable predictivity. The evidence of thyroid hormone dysregulation, sex-based differences in clinical results and dosimetry, and IVIVE predictions presented here suggest that the replacement PFECA HFPO-TeA induces a complex and toxic exposure response in rodents.

## 1. Introduction

Per- and poly-fluoroalkyl substances (PFAS) have numerous uses in both industrial applications and consumer products due to their inherent stability and ability to repel oil and water [1]. Production of perfluorooctanoic acid (PFOA) and perfluorooctane sulfonic acid (PFOS) started in the 1940s and led to accumulation in a wide variety of organisms and environmental matrices [2,3,4,5]. The chemical manufacturer 3M announced the phase out of PFOS and PFOA in 2000 [6,7]. The continued persistence of PFOA and PFOS, despite the phase out, led to several countries severely restricting the manufacture and use of both chemicals and PFAS with similar structures in the early 2000s [6,8].

**Figure 1:**
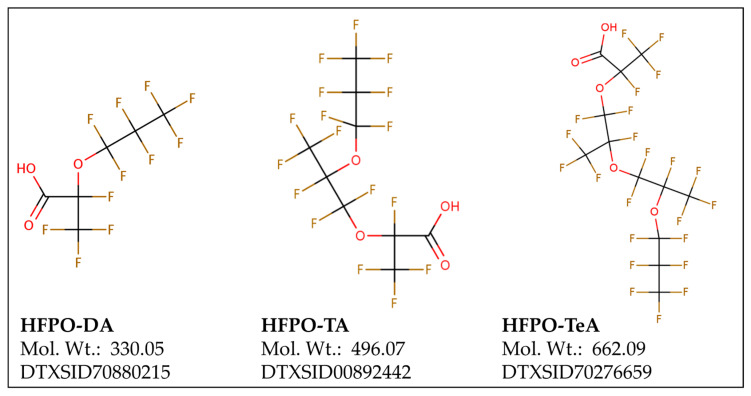
Chemical structures of hexafluoropropylene oxide (HFPO) homologues: (**Left**) HFPO-DA, (**Center**) HFPO-TA, and (**Right**) HFPO-TeA.

Manufacturers replaced PFOA, PFOS, and other structurally similar PFAS with short-chain perfluoroalkyl acids (PFAAs) and polyfluorinated compounds. These alternative PFAS are often perfluoroalkyl ether carboxylic acids (PFECAs), in which one or more oxygen atoms are inserted along the carbon backbone to aid breakdown and decomposition of the molecule [9]. The extent to which these alternative PFAS degrade is unclear [10,11]. PFECAs used as replacements for PFOA include the homologues perfluoro-2-methyl-3-oxahexanoic acid (Chemical Abstract Service Registry Number (CASRN) 13252-13-6|U.S. Environmental Protection Agency (U.S. EPA) Distributed Structure-Searchable Toxicity (DSSTox) substance identifier (DTXSID) DTXSID70880215|HFPO-DA), perfluoro-(2,5-dimethyl-3,6-dioxanonanoic) acid (CASRN 13252-14-7|DTXSID00892442|HFPO-TA), and perfluoro-(2,5,8-trimethyl-3,6,9-trioxadodecanoic) acid (CASRN 65294-16-8|DTXSID70276659|HFPO-TeA) [12] (Figure 1).

The terms HFPO-DA and GenX are often used interchangeably. GenX is a trademarked process for the production of Teflon, though it is commonly used to refer to both HFPO-DA and/or ammonium perfluoro-2-methyl-3-oxahexanoate (CASRN62037-80-3|DTXSID40108559|NH_4_-HFPO-DA), the ammonium salt of HFPO-DA [13]. They cannot be differentiated in a mass spectrometer due to their generation of the same anion, perfluoro (2-propoxypropanoate) (CASRN 122499-17-6|DTXSID70102880).

The HFPO-DA anion has been widely detected around the world, including in surface waters (max. 14 ng/L); in soils in China (max. 967 pg/g), the USA (max. 8.14 ng/g), and the Netherlands (max. 4.7 ng/g); as well as in plants (the Netherlands, 87 ng/g) [14,15,16,17,18]. The presence of HFPO-TA in surface water (max. 5.9 ng/mL) and soil (max. 0.1 ng/g) has been reported from multiple sites in China [17,18,19]. There are fewer instances of HFPO-TeA detection. One study reports on numerous organism types, with the maximum observed concentration being 3.74 ng/g dry weight in fish [20]. The main source of these PFECAs in the environment is most likely fluorochemical manufacturing. Combinations of all three homologues were detected in soil and water surrounding manufacturing plants in China as well as in fluoropolymer raw materials used for manufacturing [21,22].

To date, toxicity evaluations of PFAS, including PFOA and HFPO homologues, have provided important yet limited information. PFOA, the most widely studied of this group, is a possible human and animal carcinogen and is known to cause hepatic, reproductive, developmental, and immunological toxicities in animal models [23,24,25,26]. HFPO-DA, the most widely studied HFPO homologue, is reported to cause hepatic, hematological, immunological, renal, and reproductive toxicities in rodent studies according to a recent United States Environmental Protection Agency (U.S. EPA) Office of Water report EPA/822/R-22/005 [27].

Less is known about the toxicity of HFPO-TA and HFPO-TeA. A study by Sheng et al. demonstrated that HFPO-TA exhibits carcinogenic potential and hepatotoxicity in mice, which may be more potent than that induced by PFOA [28]. Sun et al. reported cardiotoxicity in zebrafish embryos following exposure to HFPO-TA [29]. The homologue HFPO-TeA has been associated with hepatic, cardiac, and developmental toxicity in chicken hatchlings [30]. Multiple forms of liver damage, including liver enlargement, lipid droplet accumulation, and steatosis, were observed in mice orally exposed to 1 mg/kg/day of HFPO-TeA over four weeks [31]. An increased relative liver weight was reported by Jia et al. following a seven-day exposure in CD-1 mice at concentrations up to 2 mg/kg/day [32].

Three studies have examined the impact of PFOA and the three HFPO homologues. Peng et al. demonstrated that all four compounds caused male reproductive toxicity in BALB/c mice following a 29-day exposure, with a maximum HFPO-TeA exposure concentration of 1 mg/kg/day [12]. The remaining two studies examined the binding affinity of the four compounds, reporting that HFPO-TeA has the greatest binding affinity for estrogen receptors (ERs) in zebrafish and with human fatty acid binding protein (FABP) [33,34]. The in vitro studies of HFPO homologues used dimethyl sulfoxide (DMSO) as the solvent and must be viewed cautiously based on the findings of Zhang et al., 2022, Liberatore et al., 2020, and Smeltz et al., 2023, which show significant degradation of HFPO homologues in DMSO [35,36,37].

Many PFAS, including PFOA and the three HFPO-TeA homologues, are known to perturb the PPARα signaling pathway [28,30,38]. The PPARα nuclear receptor, which is highly expressed in the liver, heart, and kidney, regulates fatty acid metabolism and peroxisome proliferation [30,31]. Disruptions of this pathway have been implicated in several downstream adverse animal health outcomes, including developmental toxicities, altered lipid and glucose metabolism, decreased plasma thyroid hormone levels, and changes in liver weight [26,27,28,29]. The full scope of the environmental and toxicological impact of these emerging PFAS remains unknown [36]. We selected HFPO-TeA for a five-day oral exposure study utilizing Sprague Dawley rats to generate toxicology data for a data-poor, emerging PFAS.

## 2. Materials and Methods

The chemicals used for solvents and additives are listed in Supplementary Text S1.

### 2.1. In Vivo Exposure Chemicals, Study Design, and Analytical Assessments

The in vivo exposure study was performed under a contract to Jacobs Technology, Inc. (Dallas, TX, USA; EP-C-15-008) and BioSpyder Technologies, Inc. (Carlsbad, CA, USA; 68HE0B20P0250). Animal handling was subcontracted out and performed by Integrated Laboratory Systems (ILS, Morrisville, NC, USA). All procedures followed Animal Welfare Act Regulations 9 CFR 1A, 1–4, and animals were handled and treated according to the Guide for the Care and Use of Laboratory Animals [39]. Male and female Sprague Dawley rats (*n* = 36/sex) were purchased from Charles River Laboratory (Raleigh, NC, USA) and allowed to acclimate for 7–10 days before initial dosing. Animals were assigned to groups using analysis of variance (ANOVA) to ensure the mean body weights of the groups were not statistically different. Same-sex pairs were housed in polycarbonate cages (23 cm × 44 cm × 21 cm) with hardwood bedding (Northeastern Products Corp., Warrensburg, NY, USA), changed twice per week. Rats were kept in rooms maintained between 20.0 and 25.0 °C and 30.0 and 70.0% humidity while exposed to a 12 h light–dark cycle. Certified Purina Pico Chow No. 5002 (Ralston Purina Co., St. Louis, MO, USA) and reverse osmosis (RO)-treated tap water (City of Durham, NC, USA) were available ad libitum. Rats were 8–10 weeks old at the time of dosing and weighed between 228 and 302 g (males) and 208 and 232 g (females).

Male and female rats (*n* = 4/sex, dose, and time point) were dosed by gavage (5 mL/kg bodyweight) once daily for five days with water (vehicle control) or HFPO-TeA at eight half-log dose levels ranging from 0.3 to 335.2 mg/kg/day (i.e., 0.3, 0.9, 2.3, 6.3, 17, 45.9, 124, and 335.2). The results reported here are part of a larger study, for which the main purpose was to use benchmark dose–response modeling of transcriptomic data to identify a gene set basis for the point of departure (POD). A number of investigators have demonstrated that a sample size of four with five or more dose levels is sufficient to detect transcriptional points of departures that are comparable to points of departures for apical effects in chronic bioassays [40,41]. Our study design also follows recommendations from the National Toxicology Program Approach to Genomic Dose–Response Modeling and Slob et al., who found that spreading the total number of animals over more dose groups results in a more accurate estimate of dose–response model parameters [42,43].

We used multiple in silico modeling approaches and a literature survey for the exposure of the HFPO homologues to refine the dose selection. The literature survey detailed exposures of HFPO-DA up to 500 mg/kg/day with no reported premature mortality [44,45]. The in silico modeling approaches included using ToxPrint structural categories (aggregated or individual categories), a 5th percentile from a distribution of PODs, and global predictive model approaches using medial no observed effect level (NOEL) and no observed adverse effect level (NOAEL) values. Structural categories were first assigned to HFPO-TeA, and aggregated POD toxicity values were derived on a per risk assessment class basis. A 5th percentile and bootstrapped confidence interval around the 5th percentile value was computed based on all POD values for all the substances. This value was adjusted based on a 100 safety factor and used as a Threshold of Toxicological Concern (TCC) for HFPO-TeA. The aggregated median POD values were used in a machine learning approach, which relied upon structural category assignment, the *n*-octanol/water partition coefficient (log K_OW_), and the chain length as descriptors. A Kth nearest-neighbor algorithm model resulted in the most promising performance out of the attempted approaches based on the coefficient of determination (R^2^) and root mean square error (RMSE). Hyperparameter tuning was conducted, and the 5-fold coefficient of variation (CV) was performed for the dataset. The model dataset was then resampled 1000 times to create 1000 models and generate predictions. The median values and their lower and upper 95% confidence values were identified. Dose levels were selected by starting at the upper 95% value and using half-log spacing.

Perfluoro(2,5,8-trimethyl-3,6,9-trioxadodecanoic) acid (HFPO-TeA) was obtained from Synquest Laboratories (Alachua, FL, USA; 98% purity) for use in generating the in vivo dosing solutions. Dosing solutions of HFPO-TeA were prepared in water (0.06, 0.18, 0.46, 1.26, 3.4, 9.2, 24.8, and 67 mg/mL). The dosing solutions were stirred for 30 min prior to dose administration to the end of dosing to ensure solution homogeneity. The purity check performed by BioSpyder prior to use by ILS indicated that the purity was 96.94%, with unidentified impurities observed at ions of *m*/*z* 262.99 ± 1.00 Da, 1524.49 ± 1.00 Da, and 517.06 ± 1.00 Da. Peak abundances relative to the HFPO-TeA peak were 0.44%, 0.03%, and 2.52%, respectively.

Clinical observations were performed prior to first administration and at termination. Moribundity and mortality were assessed twice daily on weekdays and once daily on weekends. Animals found moribund were euthanized by carbon dioxide inhalation and death was confirmed by cervical dislocation. Further data are presented only for rats from the dose groups ≤ 17 mg/kg/day.

Whole blood was collected from the retro-orbital plexus under isoflurane anesthesia 2 h following the first dose and by cardiac puncture 24 h after the last dosage. Blood was stored in anticoagulant potassium ethylenediaminetetraacetic acid (K_3_EDTA)-coated vacutainers, and then processed to isolate plasma via centrifugation. Animals were euthanized via carbon dioxide asphyxiation and subsequent exsanguination. Liver was collected, weighed, cubed, and divided into three aliquots. One aliquot of tissue was flash-frozen in liquid nitrogen and stored at −80 °C for analytical chemistry analyses. The remaining two aliquots were placed in RNALater and frozen for use in the companion transcriptomic study. Liver and plasma samples were received at the U.S. EPA Center for Computational Toxicology and Exposure, Advanced Analytical Chemistry Methods Branch in Research Triangle Park, North Carolina, USA, and stored at −80 °C prior to extraction.

#### 2.1.1. Plasma Thyroid Hormone Assessment

##### Chemicals and Extraction Procedure

Information on the chemicals used in the plasma thyroid hormone analysis can be found in Supplementary Text S2 and Table S3. Thyroid hormones T3, rT3, and T4 were extracted from 20 µL of plasma (5 days) following the procedure outlined by O’Shaughnessy et al. [46]. Briefly, plasma was spiked with internal standards ^13^C_6_-T3, ^13^C_6_-rT3, and ^13^C_6_-T4, and extracted using Evolute CX SPE plates (10 mg, 1 mL, Biotage, Charlotte, NC, USA). Extracts were eluted using a positive pressure manifold. Refer to Supplementary Text S2 for complete details of the extraction method.

##### Analytical Chemistry

Targeted, quantitative analysis of the thyroid hormone levels was conducted using a 6500+ QTRAP liquid chromatography/mass spectrometer system (Sciex, Framingham, MA, USA) operated in positive ion multiple reaction monitoring (MRM) scan mode with electrospray ionization (ESI). A Restek column (Raptor Biphenyl, 100 × 2.1 mm, particle size 2.6 µm) was used for chromatographic separation with a flow rate of 0.3 mL/min and a temperature of 50 °C. Gradient elution was obtained using eluent A (0.1% formic acid; FA (aqueous)) and eluent B (MeOH with 0.1% FA). The gradient used is found in Table S4. Additional instrument parameters can be found in Table S5 and monitored transitions in Table S6. Statistical analysis is described in Supplementary Text S3.

#### 2.1.2. Plasma HFPO-TeA Dosimetry Assessment

##### Chemicals and Extraction Procedure

Detailed information on the procedure, chemicals, and materials used in the plasma HFPO-TeA dosimetry work can be found in Supplementary Text S4 and Table S3. Plasma preparation (2 h and 5 days) was modified from Conley et al.’s work and is detailed in Supplementary Text S4 [45]. Briefly, aliquots (25 µL) were mixed with methanol (MeOH) and water (H_2_O) containing 0.1 M FA, spiked with the internal standard perfluorohexadecanoic acid (PFHxDA), and then denatured plasma proteins were precipitated using ACN. Samples were held at −20 °C then centrifuged for separation of the supernatant. Supernatants were collected and stored at −20 °C prior to analysis.

##### Analytical Chemistry

Targeted analysis of HFPO-TeA was conducted on a Sciex X500R QTOF liquid chromatography/mass spectrometer system operated in high-resolution MRM negative ion ESI mode. A Phenomenex C18 column (Kinetex XB-C18, 100 × 2.1 mm, particle size 2.6 µm) was used for chromatographic separation with the flow rate set to 0.2 mL/min and a temperature of 40 °C. Gradient elution was achieved with eluent A (95:5 H_2_O/MeOH) and eluent B (95:5 Methanol/H_2_O). Ammonium formate (4 mM) was added to both eluents. The gradient is shown in Table S7. Additional details of the instrument parameters are provided in Table S8 and monitored transitions in Table S9. Matrix blanks were analyzed with each sample set. Statistical analysis is described in Supplementary Text S3. Normalization calculations are described in Supplementary Text S5 [47,48].

#### 2.1.3. Liver HFPO-TeA Dosimetry Assessment

##### Chemicals, Materials, and Extraction Procedure

Information on the chemicals and materials used in the liver plasma HFPO-TeA dosimetry work can be found in Supplementary Text S6 and Table S3. Liver sample preparation was modified from Conley et al.’s work and detailed in Supplementary Text S6 [49]. Briefly, samples were spiked with the internal standard PFHxDA then homogenized in 200 µL of ACN containing 0.1% formic acid. The sample was centrifuged, and the supernatant was transferred. This process was repeated twice more, then stored at −20 °C prior to analysis.

##### Analytical Chemistry

Liver samples were analyzed using the same analytical assessment procedure as the plasma dosimetry samples. Matrix blanks were analyzed with each sample set. HFPO-TeA was not detected in the blanks above the limit of quantitation (LOQ) of 1.58 µM. Statistical analysis is described in Supplementary Text S3, normalization calculations are described in Supplementary Text S5 [47], and the liver-to-plasma partitioning calculation is outlined in Supplementary Text S7 [32,50,51].

### 2.2. In Vivo and In Vitro Non-Targeted Analysis Chemicals and Data Analysis

Extracts of plasma (50 µL), liver (10 mg), and hepatocyte metabolism assay media (10 µL) were analyzed by high-resolution, accurate mass non-targeted analysis (NTA) for the detection and identification of potential biotransformation products of HFPO-TeA. A mixture of ^13^C-labeled PFAS compounds (MPAC-C-ES, Wellington Laboratories, Guelph, ON, Canada) were used as tracers. Specific details for the tracer mix are provided in Table S3. Samples were extracted, as detailed above (in vivo) and below (in vitro), and then analyzed in triplicate in a randomized order on a Sciex X500R QTOF. Additional information on NTA instrumental analysis and data processing parameters is presented in Supplementary Text S8 and Tables S8 and S10 [52]. Features of interest were selected after filtering for mass defects between −0.1 and 0.1, the presence of fragment ions suggesting PFAS, and a fold change > 2 compared to the vehicle controls. A screening list of potential biotransformation products was generated using the U.S. EPA web application CTS: Chemical Transformation Simulator 1.0 and Biotransformer 3.0 [53,54]. In some instances, molecular structure predictions were performed using MetFrag [55].

### 2.3. Hepatocyte Metabolic Stability and Metabolite Formation Assay Materials, Chemicals, and Extraction

#### 2.3.1. Hepatocyte Metabolic Stability Assay Study Design, Chemicals, and Calculations

Information on the chemicals and materials used in the hepatocyte metabolic work can be found in Supplementary Text S9 and Table S3. In vitro evaluations of hepatocyte metabolic stability were performed in human and rat hepatocytes, as previously described [56]. Briefly, pooled, cryopreserved hepatocyte suspensions were thawed, assessed for viability, and then diluted in William’s medium E containing dexamethasone and cell maintenance cocktail B to achieve a cell density of 50,000 cells/100 µL. The assay was started by adding hepatocyte suspensions to wells of 96-well polypropylene plates containing an equal volume of media with 2 µM of analyte (final assay concentration: 1 µM). Plates were placed in a 37 °C cell culture incubator (5% CO_2_) on a shaker set to 200 rpm. Time points assessed were T0, T15, T30, T60, T120, and T240 min. At each time point, the plates were removed from the shaker and the incubates were crashed with an equal volume of ice-cold ACN containing 1.2% FA and 16 ng/mL of ^13^C_3_ -HFPO-DA. After chilling and centrifugation at 4122× *g*, the supernatants were transferred to new collection plates and stored at −70 °C until analysis for the parent compound on the Waters instrumentation described below. Immediately prior to analysis, samples were thawed, vortexed, and centrifuged at 4200× *g*, and then diluted 1:4 in 95:5 H_2_O/ACN with 2.5 mM of ammonium acetate. Negative controls, including no cell controls and metabolically inactivated hepatocytes, were run concurrently to assess the chemical stability over the time course. Propranolol and phenacetin were run concurrently for assessing the hepatocyte metabolic activity and assay performance.

Intrinsic clearance (Cl_int_; hepatic clearance) calculations are detailed in Supplementary Text S9 [57].

#### 2.3.2. Hepatic Metabolic Formation Assay Study Design

For the hepatic metabolite formation assays, the procedure described above was followed with the following modifications: assay concentrations of 50 and 100 µM were used, and samples were collected at times T0, T30, T60, and T120 min. Aliquots (10 µL) were combined with an equal volume of H_2_O. Non-targeted screening of potential biotransformation or degradation products of these samples was performed on the Sciex instrumentation described above.

### 2.4. Ultracentrifugation Plasma Protein Binding Assay Chemicals, Materials, Study Design, and Calculations

#### Study Design, Chemicals, and Calculations

Information on the chemicals and materials used in the plasma protein binding work can be found in Supplementary Text S10 and Table S3. Human and rodent plasma were evaluated for plasma protein binding using ultracentrifugation, as previously described [44]. Working solutions of 3 mM of HFPO-DA, HFPO-TA, and HFPO-TeA were prepared separately in ethanol, with each working solution containing 3 mM of testosterone (Sigma-Aldrich; ≥98% pure) as a reference compound for assay performance. An aliquot of each stock solution (13.3 µL) was added to separate 15 mL tubes with 3.987 mL of plasma for a final assay concentration of 10 µM. The plasma solutions were incubated at 37 °C and 125 rpm for 60 min. One-hour stability samples (T60 min) were drawn in triplicate and diluted in four volumes of plasma ultrafiltrate. Aliquots (1 mL) from each plasma solution were drawn in triplicate and transferred to polycarbonate tubing. These samples underwent ultracentrifugation (Beckman OptimaMax; Beckman Coulter Inc., Brea, CA, USA) at 850,000× *g* for 4 h at 37 °C.

The remaining plasma solutions were returned for incubation at 37 °C and 125 rpm for an additional 4 h. Five-hour stability samples (T300 min) were then drawn in triplicate and diluted in four volumes of plasma ultrafiltrate. After ultracentrifugation, the aqueous fraction (AF) was collected by transferring the supernatant to a new tube and diluting in an equal volume of plasma for matrix matching. All samples were combined with 3 volumes of ice-cold ACN containing 16 ng/mL of ^13^C_3_-HFPO-DA and 16 ng/mL of ^13^C-Testosterone (Cambridge Isotope Laboratories; 98% pure). Samples were vigorously mixed, stored for 10 min at −20 °C, and then centrifuged at 12,000× *g* for 10 min at 4 °C. Supernatants were collected then stored at −70 °C prior to analysis.

Fraction unbound (f_u_) in plasma (f_up_) and percent stability calculations are detailed in Supplementary Text S10. Hepatic clearance, the f_u_ in blood (f_ub_) (f_u_ in plasma adjusted for blood:plasma partitioning), and renal clearance were used to derive the steady-state concentration (C_ss_) of each compound that will be used for in vitro–in vivo extrapolation (IVIVE) [58,59]. Renal clearance was calculated using the glomerular filtration rate (a species-dependent constant) and the f_ub_. The C_ss_ calculation is detailed in Supplementary Text S11.

### 2.5. In Vitro Study Targeted Sample Analysis

Targeted, quantitative analysis of in vitro hepatocyte metabolic stability and plasma protein binding assay samples was conducted on a Waters Xevo-TQS micro mass spectrometer (Waters, Milford, MA, USA) ultra-high-performance liquid chromatography tandem mass spectrometry system (UPLC-MS/MS), modified with a PFAS analysis kit. Samples were analyzed in negative ion mode with UniSpray ionization. A CORTECS T3 column (Waters CORTECS, 3 × 100 mm, particle size 2.7 µm) was used for chromatographic separation with a flow rate of 0.6 mL/min and a temperature of 55 °C. Gradient elution was used with eluent A (95:5 H_2_O/ACN) and eluent B (95:5 ACN/H_2_O) with 2.5 mM of ammonium acetate. The gradient is available in Table S11. Additional details for MS parameters are found in Table S12 and monitored transitions are found in Table S13.

### 2.6. LD50 Calculation

The mortality incident data were analyzed using BMDS 3.3.2, with the dichotomous model selected as the model type [60]. The Benchmark Response was set at 0.5 extra risk. LD50 estimates were based on the model with the lowest Akaike information criterion (AIC).

## 3. Results

### 3.1. In Vivo Results

#### 3.1.1. Rat Body Weight, Liver Weights, and Clinical Observations

Body weight loss was observed over the five-day study period in the 6.3 mg/kg/day (female) and 17 mg/kg/day (both sexes) dose groups (Table 1). The average relative liver weight in males trended upward with the increasing dose between dose groups of 0.9–6.3 mg/kg/day, followed by a modest decrease for the 17 mg/kg/day dose group. This decrease is likely due to the overall body weight loss from HFPO-TeA toxic stress. The average relative liver weight in females trended upward with the increasing dose from dose levels ≥ 0.3 mg/kg/day. Data for individual rats are found in Table S14.

Clinical observations also suggest toxic responses to the HFPO-TeA exposure. Abnormal clinical observations in male rats included lethargy, piloerection, thinness, hunching, coldness to touch, abnormal breathing, and decreased movement in the 17 and 45.9 mg/kg/day dose groups. Female rats exhibited numerous abnormal clinical observations, beginning with piloerection and thinness within the 6.3 mg/kg/day dose group. In addition, coldness to touch and hunching were observed for the 17 mg/kg/day dose group females. Abnormal breathing was observed alongside previous signs of toxicity in the 45.9 mg/kg/day females and decreased movement and lethargy were noted in the 124 mg/kg/day females.

Premature death occurred in male and female rats from the 45.9, 124, and 335.2 mg/kg/day dose groups. Only one male rat in the 45.9 mg/kg/day group and none of the males in the 124 and 335.2 mg/kg/day dose groups survived past Day 1. The fourth male rat from the 45.9 mg/kg/day dose group expired on Day 4. Of the female rats, two from the 45.9 mg/kg/day dose group expired on Day 1, with the remaining two only surviving to Day 4. Three female rats from each of the 124 and 335.2 mg/kg/day dose groups expired on Day 1, and the fourth rat from the two highest dose levels died on Day 3. For both males and females, BMDS 3.3.2 analysis of the mortality data selected the multistage 3 as the best model fit, resulting in LD50 estimates (lower and upper 95% bounds) of 37.77 (26.09 and 59.96) mg/kg/day for males and 28.23 (18.69 and 41.57) mg/kg/day for females.

#### 3.1.2. Plasma Thyroid Hormones

The average concentrations of rT3 did not significantly change between sexes or between dose levels versus controls (Table 2). Average concentrations of T3 and T4 generally decreased with the increasing dose for both sexes (Table 2). Concentrations of T3 in males (17 mg/kg/day) and females (6.3 and 17 mg/kg/day) significantly decreased (*p* < 0.05) compared to vehicle controls. Both sexes saw a significant decrease (*p* < 0.05) in T4 concentrations at 17 mg/kg/day compared to the vehicle controls. Individual rat data are found in Table S15.

#### 3.1.3. Plasma HFPO-TeA Dosimetry

Average plasma HFPO-TeA concentrations following 2 h of exposure ranged from 0.137 ± 0.033 µM to 224 ± 76 µM for females and 0.150 ± 0.020 µM to 126 ± 62 µM for males across the 0.3–17 mg/kg/day dose groups (Table 3). Average plasma HFPO-TeA concentrations were 1.1–1.8 times greater in female rats after 2 h of exposure for dose groups above 0.3 mg/kg/day; however, the differences were not statistically significant between the sexes. Individual data for all rats sampled 2 h post-exposure are shown in Table S16.

**Figure 2:**
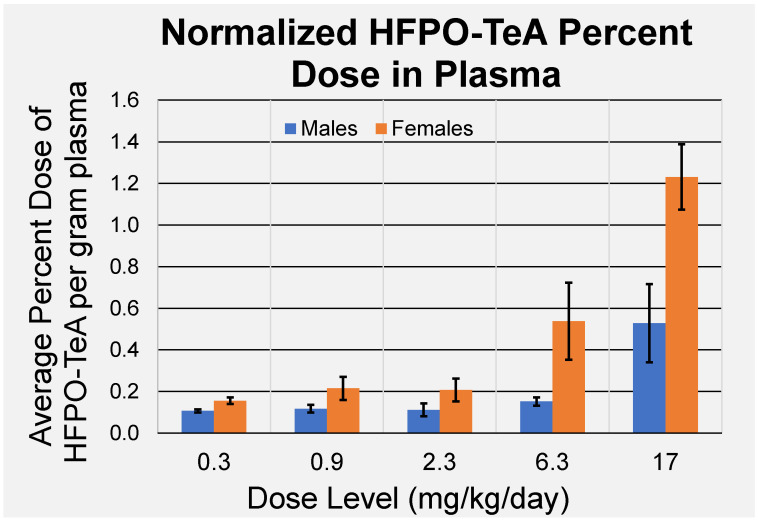
Percent dose of HFPO-TeA per gram of plasma following five days of exposure. Data points represent mean ± standard deviation (*n* = 4).

Following 5 days of exposure, average HFPO-TeA plasma concentrations were higher for female rats, ranging from 0.854 ± 0.086 µM to 278 ± 28 µM across the 0.3–17 mg/kg/day dose groups (Table 3). Concentrations in females were 1.3–2.1 times greater than those observed in males for dose groups ≥ 0.9 mg/kg/day. The only statistically significant, sex-related difference (*p* ≤ 0.05) was between females and males at 17 mg/kg/day. Individual data for all rats sampled after five days of exposure are shown in Table S17. When presented as percent dose per gram of tissue, there appears to be an increased accumulation of HFPO-TeA in blood within the 6.3 and 17 mg/g/day dose groups in females and the 17 mg/kg/day dose group in males (Figure 2).

#### 3.1.4. Liver HFPO-TeA Dosimetry

**Figure 3:**
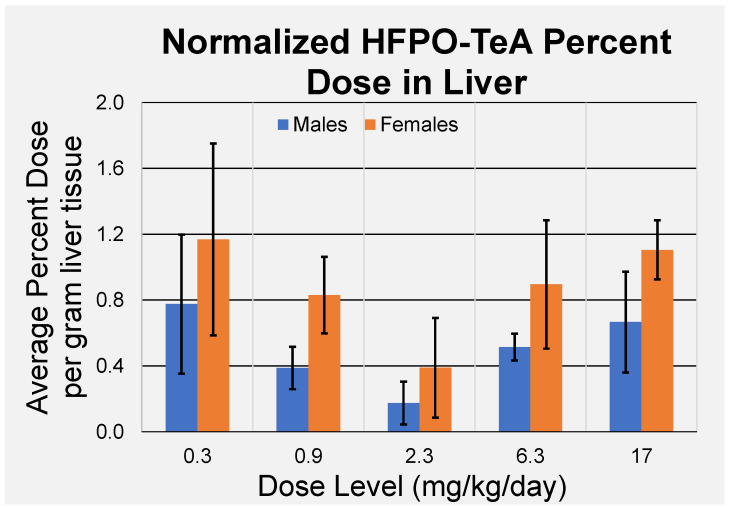
Percent dose of HFPO-TeA per gram of liver following five days of exposure. Data points represent mean ± standard deviation (*n* = 4).

Female liver concentrations ranged from 6.38 ± 3.14 µM for the 0.3 mg/kg/day dose group to 250 ± 39.3 µM for the 17 mg/kg/day dose group. Male liver concentrations ranged from 6.07 ± 3.40 µM to 210 ± 84.9 µM across the 0.3–17 mg/kg/day dose groups (Table 3). Female rats had slightly higher HFPO-TeA liver concentrations across all dose levels compared to males; however, differences between the sexes were not statistically significant. Liver concentrations increased with the dose, except at the 2.3 mg/kg/day dose, in which liver concentrations were not significantly greater than those of the 0.9 mg/kg/day dose group. In contrast to the plasma, there does not appear to be a dose-dependent sequestration in the liver at the higher dose levels for either sex (Figure 3). Individual liver concentrations for each rat can be found in Table S18.

Liver-to-plasma ratios (K_p_) were calculated for each dose level and sex, with the calculation outlined in Supplementary Text S7 (Table 4). The liver K_p_ value for male rats was 7.28 at the lowest dose, and subsequently decreased by at least half at the higher dose levels (Table 4). For female rats, K_p_ values showed a steady decrease as the exposure concentrations increased. For both sexes, K_p_ decreased seven-fold from the lowest to the highest dose.

### 3.2. Non-Targeted Analysis

#### 3.2.1. In Vivo Assays

Non-targeted analysis of plasma and liver was used to investigate the potential for HFPO-TeA biotransformation. Spectra generated from plasma and liver extracts were screened for features having fragment ions that are characteristic of the HFPO-TeA structural backbone as well as biotransformation products predicted by CTS: Chemical Transformation Simulator (Table 5) [53].

**Figure 4:**
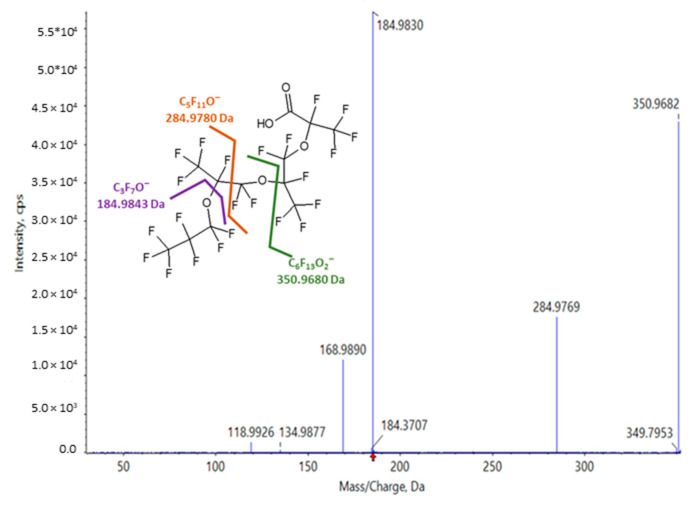
High-resolution MS/MS spectrum of the in-source ion (*m*/*z* 350.9680) of HFPO-TeA.

In the source of the mass spectrometer, HFPO-TeA ionizes to form a labile (M-H)^-^ ion that fragments to lose 310.9766 Da (C_6_HF_10_O_3_) and form a fragment ion of *m*/*z* 350.9680 (C_6_F_13_O_2_^−^). This species generates an MS/MS spectrum with fragment ions of *m*/*z* 284.9780 (C_5_F_11_O^−^), 184.9843 (C_3_F_7_O^−^), 168.9889 (C_3_F_7_^−^), 134.9876 (C_2_F_5_O^−^), and 118.9921 (C_2_F_5_^−^) that are characteristic of the HFPO-TeA backbone (Figure 4). No ions that suggested the presence of the three predicted HFPO-TeA conjugates were observed in plasma or liver with this analytical method. To assess whether other potential biotransformation products were present, peak tables generated using Sciex MarkerView from TOFMS scans of plasma and liver extracts from the 17 mg/kg/day dose group were screened for features with a negative mass defect that were not present in the vehicle controls and yielded MS/MS spectra with HFPO-TeA or PFAS characteristic ions. Several features were selected for further evaluation as ions of interest based on the filtering criteria. For each, we used the exact masses observed in TOFMS scans, MS/MS spectral features, and chromatographic retention times to inform annotation of the precursor molecules. The ions of interest, their observed fragment ions and proposed chemical formula, the matrix they were observed in, and the retention times relative to HFPO-TeA are summarized in Table 6.

**Figure 5:**
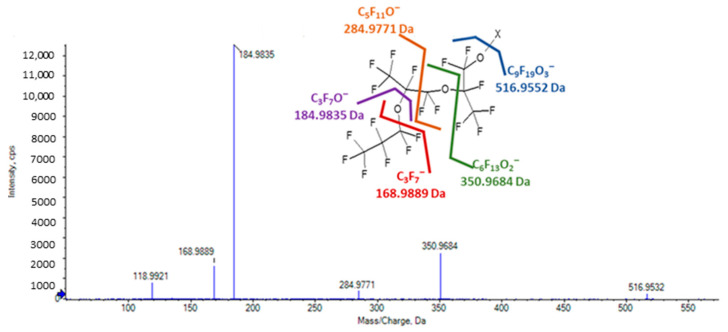
High-resolution MS/MS spectrum of the ion of *m*/*z* 516.9552, where X in the proposed structure is an unknown headgroup.

Characteristic MS/MS spectra for each precursor ion of interest were probed for clues as to the identity of the molecular species. The MS/MS spectrum shown in Figure 5 corresponds to the ion of interest *m*/*z* 516.9552. The fragment ions of *m*/*z* 350.9680, 184.9835, and 168.9889 observed in the spectrum are consistent with fragments from PFECAs and characteristic of fragmentation of the ion generated upon in-source fragmentation of the HFPO-TeA molecular species. The masses and responses relative to the base peak of the spectrum for the ion of *m*/*z* 516.9552 were entered into MetFrag for selection of potential molecular candidates. An in silico spectrum generated with MetFrag for 1,1,2,3,3,3-hexafluoro-2-[1,1,2,3,3,3-hexafluoro-2-(1,1,2,2,3,3,3-heptafluoropropoxy) propoxy]propan-1-ol (C_9_HF_19_O_3|_PubChem CID 13244841|exact mass 517.9622290 Da) matched all experimental MS/MS fragment ions (Figure 5, where X = H). The corresponding observed TOFMS mass fit the molecular formula to within 5 ppm. Such a species could be formed by O-dealkylation of HFPO-TeA to yield a 9-carbon alcohol.

**Figure 6:**
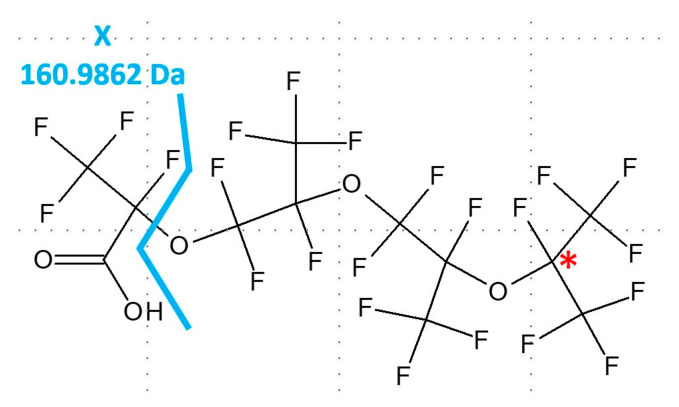
Structure of perfluoro(2,5,8,10-tetramethyl-3,6,9-trioxaundecanoic) acid (CAS 1212077-14-9|DTXSID40892441). The compound can be represented by a C_3_HF_4_O_2_ headgroup attached to a backbone structure similar to HFPO-TeA but with a branched tail (labeled with a red asterisk). The headgroup, labeled here with an X and the exact mass of the substructure, could be the unknown headgroup for the proposed structure of the ion of *m*/*z* 516.9552 shown in Figure 5.

However, the observed ion eluted after HFPO-TeA with a relative retention time of 1.07 min. A compound with 9 carbons would be expected to elute chromatographically with a relative retention time of approximately 0.87 min compared to the 12-carbon HFPO-TeA, as demonstrated by the relative retention time of the ^13^C_9_-PFNA tracer. The ion of *m*/*z* 516.9552 may be an in-source fragment of a species with an undefined head group (X) comprised of approximately 3 carbons that is produced by transformation of an HFPO-TeA precursor, or a species related to HFPO-TeA, such as the branched isomer perfluoro(2,5,8,10-tetramethyl-3,6,9-trioxaundecanoic) acid (CAS 1212077-14-9|DTXSID40892441|Figure 6). A corresponding dimer was not observed. The branched isomer does not appear to be available commercially. Since the mass of a molecular ion of the proposed compound was not observed, and a standard is not available, this tentative identification was assigned a Schymanski et al. confidence level of 5 [61]. The ion of *m*/*z* 516.9552 detected in the plasma and liver may be related to the ion of *m*/*z* 517.06 ± 1.00 detected in the HFPO-TeA stock used for the in vivo study at 2.52% relative abundance.

**Figure 7:**
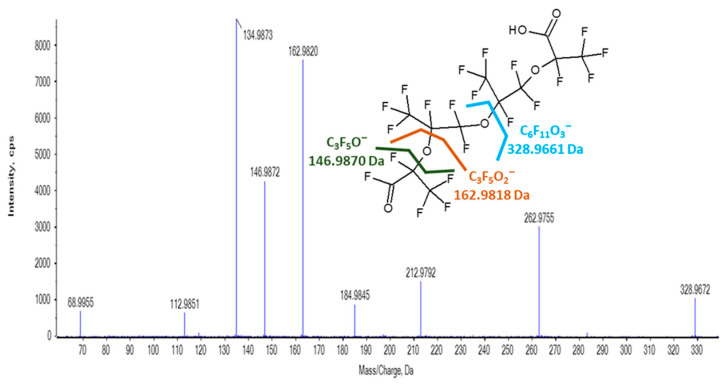
High-resolution TOF MS/MS spectrum and proposed structure of the ion of *m*/*z* 328.9661.

The MS/MS spectrum of a second ion of interest, *m*/*z* 328.9661, exhibited several fragment ions that are characteristic of HFPO-TeA and other PFECAs (Figure 7). The mass fit the molecular formula C_6_F_11_O_3_ to within 5 ppm. The ion slightly eluted before the HFPO-TeA ion with a relative retention time of 0.98 min. This suggests that it is an in-source fragment of a 12-carbon species with similar polarity to HFPO-TeA. The *m*/*z* 328.9661 ion co-elutes with ions of *m*/*z* 1278.8768 and *m*/*z* 1300.8588, which may correspond to proton-bound (2M-H^−^, where M is the molecular ion) and sodium-bound (2M-2H+Na^+^) dimers of an unknown 12-carbon compound.

In some NTA scans, we also observed a co-eluting low-abundance ion of *m*/*z* 638.9504. In addition to fitting to the mass of an expected monomer, the *m*/*z* 638.9504 ion has a mass difference of 310 Da from *m*/*z* 328.9661, the same mass difference observed for the in-source fragment of HFPO-TeA from the molecular species. The *m*/*z* 328.9661 ion of interest may be an in-source fragment of a species with the C_6_HF_10_O_3_ carboxyl moiety of HFPO-TeA attached to an ether chain, in which a terminal CF_3_ group is replaced with CHO_2_, yielding a molecular formula of C_12_HF_21_O_6_ (Figure 7). This tentative identification also could not be assigned a Schymanski confidence level since a molecular species was not observed and a compound with the proposed structure is not commercially available.

**Figure 8:**
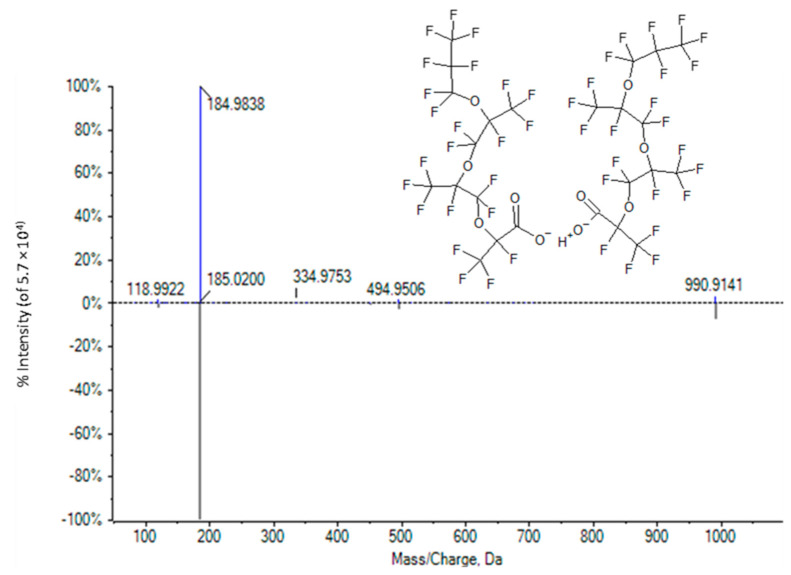
A mirror plot of: (**Top**) high-resolution TOF MS/MS spectrum of the ion of *m*/*z* 990.9106 observed only in plasma, and (**Bottom**) high-resolution TOF MS/MS spectrum of the (2M-H)^−^ dimer of HFPO-TA obtained from an authentic commercial standard.

The MS/MS spectrum of a third ion of interest, *m*/*z* 990.9106, is presented in Figure 8. The mass of the ion suggests a structure having approximately 16–20 carbons, yet the observed relative retention time to HFPO-TeA of 0.88 min indicates a species with 8–10 carbons. The low-abundance fragment ion of *m*/*z* 494.9521 is approximately half the mass of the molecular ion and fit the formula C_9_F_17_O_4_ within 5 ppm. The presence of this species is consistent with identification of the ion of *m*/*z* 990.9106 found in TOFMS scans as a ([2M-H]^−^) dimer. The co-eluting ion of *m*/*z* 1012.8940 corresponds to the (2M-2H+Na) adduct_._ The spectrum and retention time matched those for an authentic commercial standard of HFPO-TA, another PFECA oligomer of HFPO-DA (Figure 8). The library match score was 97.7. Thus, we can assign a Schymanski confidence level of 1 to this identification [61].

Peak abundances for the ions of interest were determined relative to HFPO-TeA (*m*/*z* 350.9680) for the plasma and liver for all dose groups (Table 7). The relative abundances of ions of *m*/*z* 328.9661 and *m*/*z* 516.9552 were observed to increase in plasma samples for dose groups of 0.3–6.3 mg/kg/day. The relative abundance of the ion of *m*/*z* 516.9552 for the 17 mg/kg/day dose group was within the margin of error for the abundance observed for the 6.3 mg/kg/day dose level. In liver, neither ion was observed in samples from below the 6.3 mg/kg/day dose group. In the 17 mg/kg/day dose group liver samples, the relative abundances of the ions of *m*/*z* 328.9661 and *m*/*z* 516.9552 increased by factors of more than 4 and 12, to 8.55 ± 1 and 6.25 ± 1.37, respectively. The third ion of interest corresponding to HFPO-TA, *m*/*z* 990.9106, was only observed at the highest two measured dose levels in plasma and was not detected in liver.

#### 3.2.2. In Vitro Assays

As with the in vivo assays, in vitro assays were also evaluated using NTA for the presence of potential biotransformation products and other PFAS in addition to HFPO-TeA. None of the biotransformation products predicted using CTS were observed (Table 5). In addition to the in-source fragment ion of HFPO-TeA, an ion of *m*/*z* 328.9661 was observed in all hepatocyte formation assays, including negative controls, with an average relative percent abundance of 1.02 ± 0.14 and a relative retention time of 0.98 with respect to HFPO-TeA. The presence of the ion of *m*/*z* 328.9661 at similar relative abundances in negative controls and treated hepatocytes suggests the ion is not a biologically formed product of HFPO-TeA and is likely an artifact of synthesis.

The ion of *m*/*z* 990.9106 was observed at very low abundance (<<1% relative to HFPO-TeA) in the T0 active hepatocyte preparation and not in other assay preparations. Despite the low abundance, an MS/MS spectrum that matched HFPO-TA was obtained. HFPO-TA was likely present due to the incomplete oligomerization of HFPO-DA during synthesis of HFPO-TeA.

### 3.3. In Vitro Toxicokinetics (TK) and In Vitro–In Vivo Extrapolation (IVIVE)

In vitro plasma protein binding, renal clearance, and hepatic clearance measures were derived for HFPO-TeA as well as HFPO-DA and HFPO-TA in rat and human tissues to compare TK behavior within the PFECA category and to inform cross-species comparisons (Tables S19 and S20; Figures S1–S4). All three chemicals exhibited high binding in both rat (Table S20) and human (Table S19) plasma, with the average fraction unbound in plasma (f_up_) ranging from 0.0018 to 0.0307 (spanning 17.1-fold) and 0.0013 to 0.0124 (spanning 9.5-fold), respectively (Table S21). Experimentally derived values are provided in Table 8.

No hepatic clearance was noted for any of the three PFAS evaluated in either human or rat hepatocytes (Tables S17 and S18; Figures S1 and S2). Clearance rates for the positive control compound propranolol were consistent with historical values, indicating that the hepatocyte assay was functioning properly (Figures S3 and S4).

For in vitro–in vivo extrapolation (IVIVE), the steady-state concentration (C_ss_) was calculated using Equation G detailed in Supplementary Text S11 and the values presented in Table S21. Employing an intake dosage (ID) of 1 mg/kg/day for HFPO-TeA, the C_ss_ values were predicted to be 29.34 µM and 18.96 µM in humans and rats, respectively. IVIVE values for all HFPO chemicals in rats and humans are provided in Table 8. Human HFPO-TA C_ss_ values were the highest, at 375.76 µM, assuming a 1 mg/kg/day exposure.

## 4. Discussion

In this study, we evaluated in vivo and in vitro exposure to the HFPO tetramer acid HFPO-TeA. The results for in vivo exposure provided a variety of data points for understanding the rodent response, including clinical indications of toxic stress, changes to body and liver weights, thyroid hormone dysregulation, plasma, and liver dosimetry, and additional PFAS in the exposure dose. The in vitro portion of the study enabled cross-species comparisons that provide context for human exposure.

Weight changes associated with PFAS exposure were observed for HFPO-TeA and have also been observed with other in vivo studies. Rats exposed to PFOA exhibited a decreased weight gain when dosed above 20 mg/kg/day compared to controls and lower doses. Male Sprague Dawley rats were exposed to a high (20 mg/kg/day) and a low (5 mg/kg/day) dose of PFOA over 28 days. The rats receiving the high exposure showed a significant decrease (*p* < 0.05) in average weight, whereas the low dose did not [62]. A significant decrease in weight gain (*p* < 0.05) was observed by Loveless et al. for male Sprague Dawley rats exposed to 30 mg/kg/day of PFOA for 13 days [63]. Pregnant dams orally dosed with GenX showed statistically significant differences in weight gain at the two highest dose levels of 250 mg/kg/day (*p* < 0.05) and 500 mg/kg/day (*p* < 0.001) during a five-day dosing window [45]. Our experimental data for oral HFPO-TeA exposure over five days showed weight loss in non-pregnant females occurring at dose concentrations approximately 40 and 15 times lower than the HFPO-DA exposure to pregnant dams, where weight gain was slowed. Male rats from our HFPO-TeA five-day oral exposure showed weight loss, whereas at the same approximate dose concentration, male rats exposed to PFOA by Loveless et al. and Cui et al. for longer time periods exhibited a decrease in weight gain [62,63]. The amount of weight loss observed for HFPO-TeA exposures at similar concentrations to HFPO-DA and PFOA for female and male rats, respectively, suggests that HFPO-TeA may induce more harm than HFPO-DA and PFOA in Sprague Dawley rats.

Significant increases (*p* < 0.05) were observed in relative liver weights at dose levels ≥0.9 mg/kg/day for both sexes for our oral, five-day exposure of rats to HFPO-TeA. We are not aware of other HFPO-TeA dosing studies in rats, but studies have been undertaken with mice. Jia et al. exposed male CD-1 mice to three concentrations (0.02, 0.20, and 2.00 mg/kg/day) of HFPO-TeA for seven days and reported a significant increase in relative liver weight (*p* < 0.0001) for the 2.00 mg/kg/day exposures compared to controls [32]. Male ICR mice orally exposed to 1 mg/kg/day of HFPO-TeA over four weeks had significant (*p* < 0.01) increases in relative liver weight [31]. The study also reported that mice with increased relative liver weight exhibited increased levels of PPARα proteins compared to controls, suggesting the potential that PPARα activation could be associated with an increase in relative liver weight [31]. Das et al. exposed male SV129 wild-type and PPARα-null mice to one of three PFAS: perfluorononanoic acid (PFNA), perfluorohexanesulfonate (PFHxS), or PFOA [64]. Both types of mice exposed to any of the three PFAS showed increases in absolute and relative liver weights. Thus, current data are unclear as to the contribution of PPARα in relative liver weight gain in rodents exposed to PFAS. Without further data, such as increased levels of PPARα proteins, we are currently unable to attribute the observed increased relative liver weight gain solely to PPARα activation.

Our in vivo results suggest that HFPO-TeA may perturb thyroid hormone regulation. We observed significant decreases (*p* < 0.05) in T3 and T4 concentrations vs. controls for both sexes in the 17 mg/kg/day dose group, and for T3 in females at the 6.3 mg/kg/day dose. Recently, Conley et al. observed a decrease in T4 levels in groups orally exposed to doses of 250 and 500 mg/kg/day of HFPO-DA [45]. Previous reproductive and developmental toxicity research on the legacy compound PFOA by the same authors showed that oral exposure to 10 mg/kg/day from gestation day eight to postnatal day two was associated with decreased T4 levels in dams and pups [65]. Concomitant weight loss and clinical observations of toxicity are likely to be the cause of our experimentally observed thyroid hormone changes at the 6.3 and 17 mg/kg/day dose levels.

Plasma dosimetry for this exposure study demonstrated differential responses in male and female rats. Plasma concentrations of HFPO-TeA ranged from 0.827 to 168 µM for males and 0.853 to 263 µM for females, showing a significant difference (*p* < 0.05) between the sexes only at the 17 mg/kg/day dose level (Table 3). Sex-specific responses to PFAS have previously been noted to occur in humans, mice, and rats [66,67,68,69,70]. Normalized percent dose per gram of plasma data for both sexes increased across the dose range (Figure 2). Within plasma, PFAS are known to behave in a similar manner to free fatty acids and preferentially bind strongest to albumin, followed by other lipoproteins [71,72]. We observed strong binding by the three tested HFPO homologues in the in vitro assays, with the f_up_ ≤ 0.012 and ≤0.030 in humans and rats, respectively, for all three compounds (Table 8). Based on the absence of a plateau in the normalized results, our experimental data may indicate bioaccumulative potential.

Liver concentrations of HFPO-TeA ranged from 6.07 to 210 µM for males and 6.38 to 250 µM for females (Table 3). Normalized percent dose per gram of tissue data exhibited a U-shaped trend (Figure 3). The data for the lowest three dose groups displayed a linear decrease in HFPO-TeA concentrations as the dose levels increased, suggesting potential clearance of HFPO-TeA from the liver tissue. After decreasing up to the 2.3 mg/kg/day exposure, concentrations sharply increased between the 2.3 and 6.3 mg/kg/day doses, with averages increasing seven-fold in males and five-fold in females. The observed increase with the exposure dose may suggest onset of a metabolic change that impacts the clearance mechanism for HFPO-TeA from the liver. The subsequent slight increase in liver percent dose per gram of tissue between the 6.3 and 17 mg/kg/day exposures and the decreasing Kp values with the increasing dose suggest that the system may be approaching equilibrium or metabolism could be impacted by the toxic stress evident from clinical observations.

A likely clearance mechanism for PFAS in the liver is uptake by fatty acid binding protein (FABP), as PFAS have been found to displace natural ligands from this clearance protein [73]. Dong et al. observed upregulated expression of liver FABPs in chicken hatchlings and hepatic steatosis after embryos were exposed to HFPO-TeA [26]. Interestingly, neither FABP upregulation nor hepatic steatosis occurred after in ovo silencing of PPARα. Sheng et al. also observed upregulation of FABPs in human hepatocytes exposed to HFPO-TeA [34]. In addition, their investigation of the binding affinity of eight PFAS, including PFOA, HFPO-TA, and HFPO-TeA, to human liver FABP showed HFPO-TeA to have the highest binding affinity of the group [34]. The observed decrease in normalized liver HFPO-TeA concentrations between dose levels of 0.3, 0.9, and 2.3 mg/kg/day warrants a lipidomics investigation into the possibility of potential activation of FABP, PPARα, or other transport mechanisms within this system.

Previous animal studies showed that PFAS may be highly concentrated in the blood, liver, and kidney [50,62,74]. We investigated the potential of HFPO-TeA liver accumulation by calculating K_p_ values for all dose levels and observed an approximately seven-fold decrease as the dose concentration increased. Jia et al. reported that the experimental K_p_ values for male mice exposed to HFPO-TeA at 0.02, 0.2, and 2 mg/kg/day for 7 days were approximately 20, 40, and 5, respectively [32]. Our experimental K_p_ value for the 0.3 mg/kg/day exposure group after 5 days was 6 times lower (7.28 for males and 7.81 for females) than Jia et al. reported for the 0.2 mg/kg/day exposure group. This trend is opposite from what was observed by male mice and rats orally dosed with PFOA for 28 days. Mice exposed to 2.5 mg/kg/day had a K_p_ of 1.54 [75], while rats exposed to 5 mg/kg/day had a K_p_ of 5.88 [62]. The data suggest that sequestering of different PFAS to the liver versus the plasma is partially dependent on the structure of the molecule.

We performed NTA to investigate the potential for HFPO-TeA to be biotransformed. Biotransformations of select PFAS have been demonstrated in various mammals, including humans, mice, and rats, as well as in fish, microbes, and plants [76,77,78,79,80,81]. The presence of the carboxylic acid moiety on perfluorocarboxylic acids and PFECAs suggests that they may undergo glucuronidation or other conjugation reactions [53,54]. Non-targeted analysis of plasma, liver, and hepatocyte metabolite formation assay media indicated the presence of three PFECAs, in addition to HFPO-TeA, none of which corresponded, however, to the conjugated species predicted with CTS. Since HFPO-TeA undergoes decomposition in the ion source, conjugated HFPO-TeA species may also readily decompose, and would likely not be observed as intact molecular ions. In-source fragmentation, low abundance, and lack of standards would make identification of conjugated HFPO-TeA difficult.

We did, however, observe three ions that were likely PFAS. Two of the observed ions of interest, *m*/*z* 328.9661 and *m*/*z* 516.9552, eluted later than the corresponding carbon-equivalent formulas would suggest, and appeared to be in-source fragments of higher mass species. Based on the detection of *m*/*z* 328.9661 in the hepatocyte metabolism controls and an ion of *m*/*z* 517.06 ± 1.00 in the HFPO-TeA stock used for in vivo dosing, both compounds are likely contaminants that were formed during or after the manufacturing of the HFPO-TeA standard. Both likely contaminants increased in abundance with the increasing dose relative to HFPO-TeA in plasma and liver. The results for plasma protein binding and hepatocyte clearance indicated that HFPO-TeA is strongly bound. The likely contaminants would be expected to remain at a constant ratio with respect to HFPO-TeA if their binding were similar. As both showed increasing abundance, both may experience less preferential binding than HFPO-TeA.

We can identify the third ion of interest, *m*/*z* 990.9106, as the HFPO homologue HFPO-TA with a Schymanski identification confidence level of 1 [61]. For the in vivo study, HFPO-TA was observed at low relative abundances in plasma from only two dose levels, and not in the liver. While it was not noted as a contaminant in the in vivo dosing solution, it was detected at a very low level in the active hepatocyte assay at T0. The presence of HFPO-TA is likely due to incomplete oligomerization during synthesis of HFPO-TeA and is most likely present at low levels in both in vivo and in vitro dosing solutions.

The in vitro assays demonstrated that plasma protein binding for HFPO-TA was higher than that for HFPO-TeA and HFPO-DA. With HFPO-TA observed in only two dose groups in the plasma matrix at very low abundance compared to HFPO-TeA, our in vivo data were not sufficient to make a comparison with HFPO-TeA binding. Limited toxicological data are available on HFPO-TA, but it is known to cause cardiotoxicity and developmental toxicity in zebrafish [29,82].

The in vitro component of this study provided insights into both human and rat responses to HFPO-TeA exposure. In vitro–in vivo extrapolation (IVIVE) is an approach often applied to inform dosimetry or adverse effects for chemicals lacking in vivo data. As conducted previously to describe the external dosage–internal concentration relationship, hepatic clearance, nonmetabolic clearance, and plasma protein binding were incorporated into an equation to perform the calculation [56]. When assuming a dosage of 1 mg/kg/day, the resulting C_ss_ for HFPO-TeA was estimated at 19 µM. Considering the availability of in vivo plasma concentration data 24 h after administration of 0.9 mg/kg/day, we could evaluate the predictivity of our IVIVE approach. Compared against the 2.73 μM plasma levels measured in vivo, the IVIVE predictions were seven-fold higher. For IVIVE application in chemical safety decision-making, a C_ss_ overestimation is preferred, as any subsequent administered equivalent dose estimations will err on the side of protecting human health. The overestimation in this instance is likely in part due to the inability of our hepatocyte suspension to sensitively measure the low clearance rate occurring for HFPO-TeA, and a few other simplifying assumptions in the model. Importantly, this seems to indicate that HFPO-TeA is not a substrate for renal reuptake via transporters such as OAT4. Previous IVIVE evaluations of PFOA and PFOS significantly underestimated the C_ss_ values (i.e., by >100×) due to the lack of consideration of such a mechanism [56,82].

## 5. Conclusions

In this five-day dose–response evaluation of HFPO-TeA, we used an in silico modeling approach for dose level determination and the review of dosing ranges for HFPO-DA to determine appropriate dose ranges for an initial toxicity study on the data-poor chemical. The in silico approach did not perform well, however, and led to administration of excessively high doses of HFPO-TeA and overt toxicity. Note that these dose levels were well tolerated by HFPO-DA in rats from the published literature. We recommend that tolerability assessments be conducted prior to a full animal study. The in vivo study through the 17 mg/kg/day dose level and the in vitro study provided health effects and dosimetry information that may be useful for assessing the risks associated with HFPO-TeA exposure. Compared to its homologue HFPO-DA, HFPO-TeA is more toxic. Evaluations of concentrations of HFPO-TeA in the plasma and liver demonstrated greater accumulation in plasma. The sex-specific differences noted in toxicity and toxicokinetic endpoints and the dose-dependent decrease in thyroid hormone levels point to modes of action that may warrant follow-up. Additionally, IVIVE evaluations resulted in reasonable internal dose estimations when compared to available in vivo data. In summary, this study holds value in providing the needed in vivo toxicity and toxicokinetic data for comparative evaluations with other data-poor emerging and legacy PFAS.

## Figures and Tables

**Table 1:** Average and standard deviation (*n* = 4) of body weight gain, absolute liver weights, and relative liver weights of all rats following 5 days of exposure.

Dose Level (mg/kg/Day)	Sex	Body Weight Gain (g)	Absolute Liver Weight (g)	Relative Liver Weight (g%)
0	M	31.3 ± 3.3	14.5038 ± 0.8279	4.577 ± 0.236
0.3	M	37.2 ± 9.0	15.2591 ± 1.0017	4.702 ± 0.220
0.9	M	39.6 ± 4.3	17.1421 ± 0.9748 *	5.291 ± 0.201 *
2.3	M	39.0 ± 4.8	19.2324 ± 1.2374 *	5.916 ± 0.297 *
6.3	M	31.8 ± 8.1	22.4551 ± 2.6306 *	6.983 ± 0.527 *
17	M	−51.5 ± 10.9 *	13.2446 ± 0.5851	5.600 ± 0.223 *
0	F	3.7 ± 5.7	9.6210 ± 0.7224	4.190 ± 0.189
0.3	F	6.5 ± 4.4	10.24805 ± 0.2070	4.488 ± 0.062 *
0.9	F	12.2 ± 4.3	11.8882 ± 0.5678 *	5.086 ± 0.224 *
2.3	F	12.3 ± 6.2	12.4296 ± 0.3397 *	5.296 ± 0.073 *
6.3	F	−17.8 ± 14.3 *	10.5446 ± 1.6664	5.329 ± 0.634 *
17	F	−55.2 ± 5.7 *	9.6771 ± 0.3488	5.796 ± 0.216 *

Statistically significant compared to the control (* = *p* < 0.05).

**Table 2:** Average and standard deviation (*n* = 4) of thyroid hormone concentrations (ng/mL) in plasma for all groups after 5 days of exposure. <LOQ = results were below the rT3 LOQ (0.005 ng/mL). N/A = not applicable, as there was only one replicate for that sex and dose level above the LOQ.

Dose Level (mg/kg/Day)	Sex	T3 Conc. (ng/mL)	rT3 Conc. (ng/mL)	T4 Conc. (ng/mL)
0	M	0.737 ± 0.066	0.0510 ± N/A	39.8 ± 3.6
0.3	M	0.811 ± 0.111	0.127 ± N/A	37.6 ± 10.4
0.9	M	0.723 ± 0.105	< LOQ	38.8 ± 5.2
2.3	M	0.678 ± 0.101	0.188 ± N/A	35.8 ± 10.9
6.3	M	0.630 ± 0.060	0.0847 ± 0.0257	32.9 ± 6.9
17	M	0.423 ± 0.078 *	0.0630 ± N/A	9.82 ± 2.94 *
0	F	0.870 ± 0.200	0.153 ± N/A	29.7 ± 4.3
0.3	F	0.844 ± 0.106	0.143 ± N/A	31.3 ± 3.5
0.9	F	0.799 ± 0.167	<LOQ	26.8 ± 4.2
2.3	F	0.781 ± 0.024	0.105 ± 0.012	34.9 ± 5.8
6.3	F	0.563 ± 0.162 *	0.0860 ± N/A	19.9 ± 7.1
17	F	0.585 ± 0.160 *	0.0510 ± 0.0042	15.9 ± 5.9 *

Statistically significant compared to the control (* = *p* < 0.05).

**Table 3:** Average and standard deviation (*n* = 4) of HFPO-TeA concentrations found in plasma (after 2 h and 5 days (5D) of exposure) and liver (5D of exposure). N/A = not applicable, as all sample concentrations were below the LOQs (0.0302 µM plasma and 1.58 µM liver).

Dose Level (mg/kg/Day)	Sex	2 h Plasma Conc. (µM)	5D Plasma Conc. (µM)	5D Liver Conc. (µM)
0	M	N/A	N/A	N/A
0.3	M	0.150 ± 0.020	0.827 ± 0.071	6.07 ± 3.40
0.9	M	0.573 ± 0.113	2.73 ± 0.45	9.06 ± 3.17
2.3	M	13.1 ± 2.8	6.64 ± 1.73	10.3 ± 7.5
6.3	M	34.0 ± 5.5	24.5 ± 2.3	82.7 ± 10.5 *
17	M	126 ± 62 *	168 ± 53 *	210 ± 85 *
0	F	N/A	N/A	N/A
0.3	F	0.137 ± 0.033	0.854 ± 0.086	6.38 ± 3.14
0.9	F	0.620 ± 0.133	3.62 ± 0.91	14.0 ± 4.3
2.3	F	15.1 ± 5.3	8.92 ± 2.36	16.5 ± 12.6
6.3	F	53.2 ± 16.5	52.6 ± 14.3 *	86.8 ± 31.7 *
17	F	224 ± 76 *	278 ± 28 *	250 ± 39 *

Statistically significant compared to the controls (* = *p* < 0.05).

**Table 4:** Experimental liver-to-plasma K_p_ values (average and standard deviation, *n* = 4) for all HFPO-TeA exposures.

Dose (mg/kg/Day)	Liver K_p_ (M)	Liver K_p_ (F)
0.3	7.28 ± 3.71	7.81 ± 4.68
0.9	3.38 ± 1.24	4.04 ± 1.37
2.3	1.54 ± 0.91	1.90 ± 1.47
6.3	3.41 ± 0.60	1.65 ± 0.46
17	1.32 ± 0.55 ^a^	0.903 ± 0.145

^a^ = Value is likely influenced by high toxicity and stress on the animals.

**Table 5:** Predicted biotransformation products of HFPO-TeA using CTS: Chemical Transformation Simulator. EC = Enzyme Commission.

Predicted Product	Formula	Monoisotopic Mass, Da	Metabolic Transformation	Reaction Enzyme	Biosystem
Glycine conjugate	C_14_H_4_F_23_NO_6_	718.9671	EC-based OR Phase II Transformation	Glycine-N-acyltransferase	Human
O-glucuronide	C_18_H_9_F_23_O_11_	837.9777	EC-based OR Phase II Transformation	UDP-glucuronosyltransferase	Human And Human Gut Microbial
Carnitine conjugate	C_19_H_14_F_23_NO_7_	805.0403	EC-based OR Phase II Transformation	Carnitine-O-acetyltransferase	Human

**Table 6:** Selected ions observed in TOFMS peak tables from NTA of plasma or liver from the 17 mg/kg/day dose group for both sexes. * = Retention time relative to the ion *m*/*z* 350.9680, the in-source fragment of HFPO-TeA.

Ion (*m*/*z*)	Matrix	TOF MS/MS Fragments (*m*/*z*)	TOF MS/MS Fragment Proposed Molecular Formula	Relative Retention Time (min) *
328.9661	Plasma and Liver	68.995	CF_3_^−^	0.98
		112.9852	C_2_F_3_O_2_^−^	
		134.9869	C_2_F_5_O^−^	
		146.9870	C_3_F_5_O^−^	
		162.9818	C_3_F_5_O_2_^−^	
		184.9835	C_3_F_7_O^−^	
		212.9787	C_4_F_7_O_2_^−^	
		262.9750	C_5_F_9_O_2_^−^	
350.9680	Plasma and Liver	118.9921	C_2_F_5_^−^	1.00
		134.9876	C_2_F_5_O^−^	
		168.9889	C_3_F_7_^−^	
		184.9843	C_3_F_7_O^−^	
		284.9780	C_5_F_11_O^−^	
516.9552	Plasma and Liver	118.9921	C_2_F_5_^−^	1.07
		168.9889	C_3_F_7_^−^	
		184.9835	C_3_F_7_O^−^	
		284.9771	C_5_F_11_O^−^	
		350.9680	C_6_F_13_O_2_^−^	
990.9106	Plasma	184.9833	C_3_F_7_O^−^	0.88
		494.9521	C_9_F_17_O_4_^−^	

**Table 7:** Average and standard deviation (*n* = 8) relative peak area abundance (RA) for ions of interest vs. HFPO-TeA in plasma and liver after 5 days of exposure across all dose levels. N/A = not applicable, as the ion of interest was not detected above the LOD (signal-to-noise ratio ≥ 3).

HFPO-TeA Dose Conc. (mg/kg/Day)	*m*/*z* 328.9661 RA Plasma	*m*/*z* 328.9661 RA Liver	*m*/*z* 516.9552 RA Plasma	*m*/*z* 516.9552 RA Liver	*m*/*z* 990.9106 RA Plasma
Vehicle	N/A	N/A	N/A	N/A	N/A
0.3	0.69 ± 0.34	N/A	0.09 ± 0.14	N/A	N/A
0.9	1.35 ± 0.26	N/A	0.51 ± 0.17	N/A	N/A
2.3	2.14 ±0.41	N/A	0.71 ± 0.83	N/A	N/A
6.3	3.40 ± 0.69	1.91 ± 0.43	3.58 ± 1.07	0.52 ± 0.43	0.05 ± 0.04
17	6.43 ± 0.48	8.55 ± 1.01	2.67 ± 0.61	6.25 ± 1.37	1.83 ± 0.85

**Table 8:** In vitro–in vivo extrapolation to estimate C_ss_.

Cell Type	Compound Name	Experimental f_up_	f_ub_	Cl_renal_ (L/h)	Cl_hep_ (L/h)	C_ss_ (µM)
Human	HFPO-DA	0.0098	0.0178	0.1192	0	74.75
	HFPO-TA	0.0013	0.0024	0.0158	0	375.76
	HFPO-TeA	0.0124	0.0226	0.1514	0	29.34
Rat	HFPO-DA	0.0307	0.0547	0.0044	0	7.27
	HFPO-TA	0.0018	0.0032	0.0003	0	81.84
	HFPO-TeA	0.0059	0.0105	0.008	0	18.96

Abbreviations: f_up_: fraction unbound in plasma; f_ub_: fraction unbound in blood; Cl_renal_: renal clearance; Cl_hep_: hepatic clearance; C_ss_: steady-state concentration (plasma).

## Data Availability

Considering the funding of this effort by the US EPA and in compliance with the US EPA Public Access policy, the accepted, non-formatted version of the accepted manuscript and any associated data files will be made available on PubMed Central one year after acceptance by the journal.

## Supplementary Materials

The supporting information can be downloaded at: https://www.mdpi.com/article/10.3390/toxics11120951/s1. Text S1: Chemicals; Text S2: Thyroid Hormone Chemicals and Analysis; Text S3: In Vivo Statistics; Text S4: Plasma Dosimetry Chemicals, Materials, and Analysis; Text S5: Normalization of Dosimetry Data Calculations; Text S6: Liver Dosimetry Chemicals, Materials, and Analysis; Text S7: Liver-to-Plasma Partitioning Calculations; Text S8: Non-Targeted Analysis Method and Data Processing; Text S9: Hepatocyte Metabolic Stability Assay Materials, Chemicals, and Calculations; Text S10: Plasma Protein Binding Materials, Chemicals, Assay Design, and Calculations; Text S11: In Vitro–In Vivo Extrapolation (IVIVE) Calculations. Table S1: Data processing parameters used with Sciex OS 3.0; Table S2: Data processing parameters used with Sciex MarkerView 1.3.1; Table S3: Chemical identification, vendor, purity, and experiment usage for all analytes and internal standards; Table S4: Mobile phase gradient for targeted analysis of thyroid hormones in plasma on a Sciex 6500+ QTRAP. Both mobile phases contained 0.1% formic acid as an additive; Table S5: Various instrument conditions for plasma thyroid hormone quantitation on a Sciex 6500+ QTRAP; Table S6: Monitored transitions for analysis of thyroid hormones and ^13^C-labeled internal standards on a Sciex 6500+ QTRAP. All ions were acquired in positive ion mode; Table S7: Mobile phase gradient for targeted analysis of HFPO-TeA on a Sciex X500R QTOF/MS. Both mobile phases contained ammonium formate (4 mM) as an additive; Table S8: Various instrument conditions for sample analysis on a Sciex X500R QTOF/MS; Table S9: Monitored transitions for analysis of HFPO-TeA using PFHxDA as an internal standard on a Sciex X500R QTOF/MS. The ion of *m*/*z* 350.97 is the in-source fragment formed from the HFPO-TeA molecular ion of *m*/*z* 660.97. All ions were acquired in negative ion mode; Table S10: Mobile phase gradient for non-targeted analysis on a Sciex X500R QTOF/MS. Ammonium formate (4 mM) was present in both mobile phases as an additive; Table S11: Mobile phase gradient for targeted analysis of HFPO-TeA on a Waters Xevo-TQS. Both mobile phases contained the additive ammonium acetate (2.5 mM); Table S12: Various instrument conditions for hepatocyte clearance and protein plasma binding assays on a Waters Xevo-TQS; Table S13: Monitored transitions for analysis of the in vitro analytes using a Waters Xevo-TQS; Table S14: Individual body weights, absolute liver weights, and relative liver weights for all rats after 5 days of exposure; Table S15: Individual concentrations for plasma T3, rT3, and T4 in all rats after 5 days of exposure to HFPO-TeA. N/A = Calculation not completed due the majority of samples being below the LOQ; Table S16: Individual HFPO-TeA plasma and plasma extract concentrations for all rats after 2 h of exposure. <LOQ = sample concentration was below the LOQ (20 ng/mL or 0.0302 µM), N/A = not applicable due to data being below the LOQ; Table S17: Individual HFPO-TeA plasma and plasma extract concentrations for all rats after 5 days of exposure. <LOQ = sample concentration was below the LOQ (20 ng/mL or 0.0302 µM), N/A = not applicable due to data being below the LOQ; Table S18: Individual HFPO-TeA liver (wet weight, ww) and liver extract concentrations for all rats after 5 days of exposure. <LOQ = sample concentration was below the LOQ (0.99 ng/mg ww or 1.58 µM), N/A = not applicable due to data being below the LOQ; Table S19: Experimental human TK data and calculations; Table S20: Experimental rat TK data and calculations; Table S21: IVIVE and C_ss_ calculations. Figure S1: Hepatic clearance of HFPO-DA, HFPO-TA, and HFPO-TeA over time in suspended human hepatocytes. Plotted as natural log of concentration over time and used to calculate intrinsic clearance; Figure S2: Hepatic clearance of reference compounds phenacetin and propranolol over time in suspended human hepatocytes. Plotted as natural log of concentration over time and used to calculate intrinsic clearance; Figure S3: Hepatic clearance of HFPO-DA, HFPO-TA, and HFPO-TeA over time in suspended rat hepatocytes. Plotted as natural log of concentration over time and used to calculate intrinsic clearance; Figure S4: Hepatic clearance of reference compounds phenacetin and propranolol over time in suspended rat hepatocytes. Plotted as natural log of concentration over time and used to calculate intrinsic clearance.
