# Supplementary material for: Dose Response, Dosimetric, and Metabolic Evaluations of Replacement PFAS Perfluoro-(2,5,8-trimethyl-3,6,9-trioxadodecanoic) Acid (HFPO-TeA)
Source: Toxics. 2023 Nov 22;11(12):951. doi: 10.3390/toxics11120951 (PMC10747602; doi:10.3390/toxics11120951)
Supplement: Supplementary file 1 [file toxics-11-00951-s001.zip › Renyer etal_HFPOTeA_SI Tables and Figures_Edited.pdf]

Dose-Response, Dosimetric, and Metabolic Evaluations of Replacement PFAS Perfluoro-(2,5,8-trimethyl-3,6,9-trioxadodecanoic) acid (HFPO-TeA)

Aero Renyer, Krishna Ravindra, Barbara A. Wetmore, Jermaine L. Ford, Michael DeVito, Michael F. Hughes, Leah C. Wehmas, and Denise K. MacMillan

Supporting Information

Supporting Text S1: Chemicals

Chemicals used for solvent and mobile phases include methanol (MeOH), water (H_2O), and acetonitrile (ACN) which were LC/MS grade and purchased from Honeywell Burdick & Jackson (Charlotte, NC, USA). Ethanol (EtOH) used as a solvent was ACS grade and purchased from Honeywell Burdick & Jackson. Ammonium formate (BioUltra purity) and ammonium acetate (99% pure) were used as mobile phase additives and obtained from Millipore Sigma (St. Louis, MO, USA). Formic acid (FA; 97.5% pure) was also used as an additive; it was purchased from Honeywell Fluka (Charlotte, NC, USA). Detailed information for chemicals used for each analysis may be found in Table S1.

Supporting Text S2: Thyroid Hormone Chemicals and Analysis

3,3′,5-Triiodothyronine (Chemical Abstract Service Registry Number (CASRN) 6893-02-3 ǀ U.S. Environmental Protection Agency (U.S. EPA) Distributed Structure-Searchable Toxicity (DSSTox) substance identifier (DTXSID) DTXSID8023216 ǀ T3; > 99% pure), mass-labeled 3,3′,5-triiodothyronine hydrochloride ($^{13}C_6$-T3; > 98% pure), 3,3′,5′-triiodothyronine (CASRN 5817-39-0 ǀ DTXSID3046908 ǀ rT3; > 98% pure), mass-labeled 3,3′,5′-triiodothyronine hydrochloride ($^{13}C_6$-rT3; > 99% pure), L-thyroxine (CASRN 300-30-1 ǀ DTXSID0023662 ǀ T4; > 98% pure), and mass-labeled L-thyroxine ($^{13}C_6$-T4; > 98% pure) were purchased from Cerilliant Corporation (Round Rock, Texas, USA).

Briefly, a 20 µL aliquot of plasma from each sample was loaded into individual wells of a 96-well collection plate. They were spiked with 5 µL of a 40 ng/mL $^{13}C_6$-T3, $^{13}C_6$-rT3 and $^{13}C_6$-T4 mixed solution followed by 1N hydrochloric acid (HCl) (20 µL) (36.5-38%, Fisher Scientific. Waltham, MA, USA), H_2O (100 µL), and a 50:50 H_2O/ACN solution (vol/vol) (60 µL) containing 0.1% formic acid (FA). Samples were vortexed, incubated at 37 °C for 2 hours, and brought to room temperature. They were diluted with an aqueous 0.1% acetic acid solution (LC/MS grade, Honeywell Fluka) and vortexed. The SPE well plates (Evolute CX, 96-well SPE plate, 10 mg, 1 mL, Biotage, Charlotte, NC, USA), processed with a positive pressure manifold, were conditioned with methanol followed by an aqueous 0.1% acetic acid solution prior to sample loading with low pressure. Plate wells were washed with 0.1% acetic acid followed by methanol. Thyroid hormones were eluted into a collection plate with 2.5% ammonium hydroxide (NH_4OH) (28-30%, Thermo Fisher Scientific, Waltham, MA, USA) in methanol. Extracts were then evaporated to dryness with nitrogen using a Turbovap (Biotage) then reconstituted in 100 µL of 25:75 ACN/H_2O (vol/vol) with 0.1% acetic acid. Extracts were stored in amber micro-sampling vials (Agilent Technologies, Santa Clara, CA, USA) prior to instrumental analysis.

Samples were quantitated against matrix-matched calibration curves containing a minimum of 5 points spanning the range 0.005 – 25.00 ng/mL. Calibration standards were made by combining T3, rT3, and T4 analytes with the $^{13}C_6$-T3, $^{13}C_6$-rT3, and $^{13}C_6$-T4 internal standards then with commercial rat plasma. The standards were extracted using the above procedure. Calibration curves had a quadradic fit with a correlation coefficient ≥ 0.995. Matrix blanks were analyzed with each sample set and were below the limit of quantitation, 0. 005 ng/mL plasma.

Supporting Text S3: *In Vivo* Statistics

Supporting Information

All in vivo results values are reported as mean ± one standard deviation (SD). Statistical evaluation of in vivo data was conducted using a two-way analysis of variance (ANOVA) and Dunnett's Test, both at α = 0.05 significance level, on GraphPad Prism v9.5.1.

Supporting Text S4: Plasma Dosimetry Chemicals, Materials, and Analysis

Sprague Dawley rodent plasma (pooled, mixed sex) collected with ethylenediaminetetraacetic acid (EDTA) and sterile filtered (0.2 µm) was obtained from BioIVT (Westbury, NY, USA) and stored at -80 °C until use as the control matrix for generating calibration curves and as the matrix blank during plasma sample analysis. The HFPO-TeA (98.97% pure) used for calibration curve preparation was obtained from Oakwood Products Inc. (West Columbia, South Carolina, USA). Unlabeled perfluorohexadecanoic acid (CASRN 67905-19-5 | DTXSID1070800 | PFHxDA; ≥ 98% pure) was obtained from Cambridge Isotope Laboratories (Tewksbury, MA, USA) to serve as the internal standard since a labeled HFPO-TeA standard was not commercially available.

Aliquots (25 µL) were denatured with MeOH and H_2O containing 0.1 M FA and then crashed using ACN. Volumes of MeOH, FA, and ACN were determined by the anticipated sample concentrations of HFPO-TeA: 20-1,338 ng HFPO-TeA/mL plasma = 5 µL MeOH + 100 µL FA + 500 µL ACN; 491- 80,280 ng HFPO-TeA/mL plasma = 100 µL MeOH + 875 µL FA then 50 µL subsample removed and crashed with 950 µL ACN. Anticipated sample ranges were intentionally overlapped to account for HFPO-TeA concentration differences due to biological variability among the dose groups. The internal standard PFHxDA was added prior to the ACN crash. Samples were vortexed after the addition of each solvent. After the addition of all solvents, samples were stored at -20 °C for 30 min then centrifuged at 25,000 × g for 30 min. Supernatants were collected and stored at -20 °C prior to analysis.

Samples were quantitated against matrix-matched calibration curves containing a minimum of 5 points spanning the range 20 – 80,280 ng/mL plasma. Calibrants were prepared by spiking commercial rat plasma (BioIVT) with HFPO-TeA and PFHxDA and then extracted in the same manner as study samples. Calibration curves had a quadradic fit with a correlation coefficient ≥ 0.995.

Supporting Text S5: Normalization of Dosimetry Data Calculations

Percent dose of HFPO-TeA in plasma was calculated using the observed plasma concentrations and the average plasma volume for Sprague Dawley rats [1]. The density of plasma was then used to convert from mL plasma to grams plasma for ease of comparison to the liver percent dose data [2]. Average percent dose of HFPO-TeA in liver was calculated using the observed liver concentrations and liver weights. These calculations allow for a comparison of the potential saturation of available proteins in both matrices.

Supporting Text S6: Liver Dosimetry Chemicals, Materials, and Analysis

Sprague Dawley rodent liver (unidentified, mixed sex) was obtained from BioIVT and stored at -80 °C until use as the matrix substitute for generating calibration curves and as the matrix blank during sample analysis. HFPO-TeA used for calibration curve preparation and the internal standard PFHxDA were the same as for the plasma dosimetry.

Sample aliquots (10 mg) were placed into 2 mL lysing tubes containing 1.4 mm ceramic beads (MP Biomedicals, Irvine, CA, USA) and then combined with 200 µL of ACN containing 0.1% FA and 10 ng of PFHxDA. Samples were homogenized using an OMNI Bead Ruptor (OMNI International, Kennesaw, GA, USA) with the following settings: 2 cycles, 60 second cycle time, 5.5 m/sec speed, and 30 second dwell time. The sample was centrifuged at 26,000 x g for 1 hour, the supernatant transferred to a separate

tube, and then further crashed with 200 µL ACN. This was repeated twice more to generate a final combined supernatant volume of 600 µL. Supernatants were stored at -20 °C prior to analysis.

Samples were quantitated against matrix-matched calibration curves containing a minimum of 5 points spanning the range 0.99 – 141 ng/mg liver (wet weight, ww). For the calibration standards, commercial rat liver (BioIVT) was spiked with HFPO-TeA and PFHxDA, homogenized, and extracted using the above procedure. Calibration curves had a quadradic fit with a correlation coefficient ≥ 0.995.

Supporting Text S7: Plasma-to-Liver Partitioning Calculations

Concentrations of HFPO-TeA in liver and plasma were used to calculate experimental K_P values for both sexes across all dose levels using Equation A [3-5].

$$(\mathbf{A})\ \mathrm{K}_p = \frac{Concentration_{organ}}{Concentration_{plasma}}$$

Supporting Text S8: Non-Targeted Analysis Method and Data Processing

Each sample batch included a mobile phase blank and solvent blank to monitor for the presence of laboratory contamination, and a system suitability sample consisting of the set of isotopically labeled PFAS tracer compounds spiked into extraction solvent to monitor instrument performance prior to sample analysis. The mass spectrometer was operated in negative ion ESI mode. Eluents and the column used were the same as for targeted analysis. The LC gradient used is found in Table S8. Non-targeted data were collected using both information dependent analysis (IDA) and data independent analysis (sequential window acquisition of all theoretical ions; SWATH) modes. Mass calibration was verified to within 2 ppm daily before analysis and after every 5 injections. Instrument conditions are presented in Table S6. Data processing, library searching, and formula finding were performed using Sciex software package OS 3.0 with details outlined in Table S1. A high-resolution, exact mass spectral library created in house from IDA scans of commercially available PFAS standards analyzed on the same mass spectrometer as the samples and using the same instrument parameters was used for library searches. Additionally, peak picking and alignment, normalization and statistical analysis were performed with Sciex MarkerView 1.3.1 with details outlined in Table S2. The most likely ratio (MLR) method was used for normalization of the peak abundances of sample replicates before normalization between sample groups [6].

Table S1: Data processing parameters used with Sciex OS 3.0.

Parameter	Value
Workflow	Non-Targeted Screening
Integration Algorithm	MQ4
Library Searching Algorithm	Smart Confirmation Search
Library	In House PFAS
Precursor Mass Tolerance, Da	0.4
Fragment Mass Tolerance, Da	0.4
Formula Finder Mass Tolerance, ppm	5
Formula Finder Compound Type	Man-Made
Peak Detection Minimum Retention Time	1.5
Peak Detection Sensitivity	Exhaustive

Area Ratio Threshold (Unknown/Control)	2
Group Peaks by Adduct or Charge	Yes

Table S2: Data processing parameters used with Sciex MarkerView 1.3.1.

Process	Parameter	Value
Peak Peaking	Experiment	TOFMS
	Minimum Retention Time, min	1
	Subtraction Offset, scans	10
	Subtraction Multiplication Factor	1.3
	Noise Threshold	2
	Minimum Spectral Peak Width, Da	0.01
	Minimum Retention Time Peak Width, scans	2
Alignment and Filtering	Perform Background Subtraction	Yes
	Chemical Noise Intensity Multiplier	1.5
	Maximum Number of Peaks	5000
	Intensity Threshold	2
	Isotope Filtering	No
	Remove Peaks in <, Samples	2
	Retention Time Correction	Yes
	Correction Type	Linear
	Mass Tolerance	5 ppm
	RT Tolerance, min	0.5

Supporting Text S9: Hepatocyte Metabolic Stability Assay Materials, Chemicals, and Calculations

Pooled human and rat cryopreserved primary hepatocyte suspensions were both obtained from BioIVT, a US Food and Drug Administration-licensed and inspected donor center, and produced using non-transplantable tissue. The human 50-donor pool selected from BioIVT's commercially available, pre-pooled lots was confirmed to have 85% post-thaw viability on the day of the experiment using trypan-blue exclusion. The rodent suspension was comprised of a 24-donor pool of mixed sex Sprague Dawley rat hepatocytes and was confirmed to have 78% viability on the day of the experiment. Vendor-generated metabolic characterization information was reviewed for both lots and deemed acceptable prior to study start. William's E media, dexamethasone (98% pure), and cell maintenance cocktail B were obtained from Thermo Fisher Scientific (Waltham, MA, USA), the OptiThaw hepatocyte kit from Sekisui/Xenotech (Tokyo, Japan), and the trypan blue s (Sekisui/ Xenotech, Tokyo, Japan) and the trypan blue solution from Bio-Rad (Hercules, CA, USA).

The chemicals HFPO-DA (98.97% pure), HFPO-TA (97% pure), and HFPO-TeA (94.8% pure) used for assays were procured through US EPA contract #68HE0D18D0001 with Evotec Inc. (Branford, CT, USA) which provided dosing solutions solubilized in 95% ethanol. Mass-labeled $^{13}C_3$-HFPO-DA (> 98% pure) was obtained from Wellington Laboratories and served as an internal standard. Propanolol (≥ 98%

pure) from Millipore Sigma and phenacetin (≥ 98% pure) from Sigma Aldrich were used as assay reference compounds.

Hepatic metabolic clearance data was plotted in semi-log format (ln concentration vs. time) with three replicates at each time point as previously described [7]. Linear regression analysis in conjunction with a standard F-test was used to determine whether the slope of the line (indicative of chemical clearance) was significantly different from 0. Equations (B) and C) described below were used to calculate chemical half-life (T ½) and intrinsic clearance (Cl_{int}) with units of µL/ (minute*million hepatocytes). In equation (D), the scalar 2000 is used to adjust assay cell number up to be consistent with units of 1 million cells in the Cl_{int} equation.

$$(\mathbf{B})\ k = -(slope)$$

$$(\mathbf{C})\ T_{\frac{1}{2}} = \frac{0.693}{k}$$

$$(\mathbf{D})\ Cl_{int} = \frac{(2000 * 0.693)}{T_{\frac{1}{2}}}$$

Supporting Text S10: Plasma Protein Binding Materials, Chemicals, Assay Design, and Calculations

Human plasma and Sprague Dawley rodent plasma were obtained from BioIVT. Human plasma was obtained from de-identified donors (5 male, 5 female) ranging in age from 20-50 years and pooled from both sexes. Because this analysis uses pooled, de-identified plasma, it was judged not to constitute human subjects research and therefore was not subject to IRB review or approval. Rat plasma was of mixed sex. All plasma was collected with K_3EDTA and sterile filtered (0.2 µm).

The chemicals HFPO-DA, HFPO-TA, HFPO-TeA, and $^{13}C_3$-HFPO-DA used for ultracentrifugation assay were the same as those used for the metabolic stability and metabolic formation assays.

Fraction unbound (f_u) in plasma (f_{up}) was calculated by dividing the aqueous fraction (AF) concentration by the T300 min concentration (Equation E). Chemical stability in plasma was assessed using the T60 min and T300 min concentrations (Equation F).

$$(\boldsymbol{E})\ f_{up} = \frac{AF}{T300minutes}$$

$$(\boldsymbol{F})\ Percent\ Stability = \frac{T300minutes}{T60minutes} * 100$$

Supporting Text S11: In Vitro-In Vivo Extrapolation (IVIVE) and Administered Equivalent Dose (AED) Calculations

The pharmacokinetic equation used to estimate expected steady-state concentrations (C_{ss}) is based on zero order uptake of a daily dose from the gut (assuming 100% bioavailability) with both nonmetabolic renal clearance (Cl_{renal}) and hepatic clearance ($Cl_{hepatic}$) (Equation G) [8,9]. The chemical input rate (ko) is the product of the intake dosage and the model body weight; this represents the numerator of Equation G. The Cl_{renal} calculation is shown in the first part of the denominator of Equation G; it is product of the species-dependent glomerular filtration rate (GFR) and the unbound fraction in

blood (f_{ub}) of the parent compound. The $Cl_{hepatic}$ calculation is shown in the second part of the denominator of Equation G; it is the product of the species dependent liver blood flow constant, the f_{ub} of the parent compound, and the experimentally derived intrinsic clearance (shown in Equation D) divided by the sum these three values. All values used in Equation G are for first order conditions of metabolism in the liver. All calculations, scalars, and species dependent values used to conduct the C_{ss} calculation to facilitate in vitro-in vivo extrapolation (IVIVE) are provided Table S19 within the Excel worksheet, the tab labelled IVIVE-Css.

$$(\mathbf{G})\ C_{ss} = \frac{ko}{GFR * f_u + \frac{Q1 * f_{ub} * Cl_{int}}{Q1 + f_{ub} + Cl_{int}}}$$

Based on the principle of reverse dosimetry, the C_{ss} values derived from IVIVE will be used as conversion factors to generate AEDs according to Equation H. In this equation, the AED is linearly related to the in vitro concentration and inversely related to the C_{ss}. It is only valid for first-order metabolism that is expected at ambient exposure levels.

$$(\mathbf{H})\ AED\left(\frac{mg}{\frac{kg}{day}}\right) = \frac{in\ vitro\ concentration\ (\mu M)}{C_{ss}\ (\mu M) * 1\frac{mg}{\frac{kg}{day}}}$$

Table S3. Chemical identification, vendor, purity, and experiment usage for all analytes and internal standards.

Chemical Name	CASRN	DTXSID	Vendor	Purity	Experiment
Perfluoro (2,5,8-trimethyl-3,6,9-trioxadodecanoic) acid (HFPO-TeA)	13252-13-6	70276659	Synquest Laboratories	96.94%	in vivo dosing
			Oakwood Products Inc.	98.97%	Internal dose and NTA
			Oakwood Products Inc.*	94.8%	In-vitro assays
Perfluoro (2,5-dimethyl-3,6-dioxanonanoic) acid (HFPO-TA)	13252-14-7	00892442	Matrix Scientific*	97%	In-vitro assays
Perfluoro (2-methyl-3-oxahexanoic) acid (HFPO-DA; GenX)	65294-16-8	70880215	Oakwood Products Inc.*	98.97%	In-vitro assays
$^{13}C_3$-HFPO-DA	N/A	N/A	Wellington Laboratories	> 98%	In-vitro assays
Perfluorohexadecanoic acid (PFHxDA)	67905-19-5	1070800	Cambridge Isotope Laboratories	≥ 98%	Analytical chemistry
$^{13}C_4$ Perfluorobutanoic acid ($^{13}C_4$-PFBA)	N/A	N/A	Wellington Laboratories	> 98%	Analytical chemistry
$^{13}C_5$ Perfluoropentanoic acid ($^{13}C_5$-PFeBA)	N/A	N/A	Wellington Laboratories	> 98%	Analytical chemistry
$^{13}C_5$ Perfluorohexanoic acid ($^{13}C_5$-PFHxA)	N/A	N/A	Wellington Laboratories	> 98%	Analytical chemistry
$^{13}C_9$ Perfluorooctanoic acid ($^{13}C_8$-PFOA)	N/A	N/A	Wellington Laboratories	> 98%	Analytical chemistry
$^{13}C_9$ Perfluorononanoic acid ($^{13}C_9$-PFNA)	N/A	N/A	Wellington Laboratories	> 98%	Analytical chemistry
$^{13}C_6$ Perfluorodecanoic acid ($^{13}C_6$-PFDA)	N/A	N/A	Wellington Laboratories	> 98%	Analytical chemistry
$^{13}C_7$ Perfluoroundecanoic acid ($^{13}C_7$-PFUnDA)	N/A	N/A	Wellington Laboratories	> 98%	Analytical chemistry
$^{13}C_2$ Perfluorododecanoic acid ($^{13}C_2$-PFDoDA)	N/A	N/A	Wellington Laboratories	> 98%	Analytical chemistry
$^{13}C_2$ Perfluorotetradecanoic acid ($^{13}C_2$-PFTeDA)	N/A	N/A	Wellington Laboratories	> 98%	Analytical chemistry
$^{13}C_3$ Perfluorobutane sulfonate ($^{13}C_3$-PFBS)	N/A	N/A	Wellington Laboratories	> 98%	Analytical chemistry
$^{13}C_3$ Perfluorohexane sulfonate ($^{13}C_3$-PFHxS)	N/A	N/A	Wellington Laboratories	> 98%	Analytical chemistry
$^{13}C_3$ Perfluorooctane sulfonate ($^{13}C_3$-PFOS)	N/A	N/A	Wellington Laboratories	> 98%	Analytical chemistry
3,3′,5-Triiodothyronine (T3)	6893-02-3	8023216	Cerilliant Corporation	> 99%	Analytical chemistry
$^{13}C_6$-T3	N/A	N/A	Cerilliant Corporation	> 98%	Analytical chemistry
3,3′,5′-Triiodothyronine (rT3)	5817-39-0	3046908	Cerilliant Corporation	> 98%	Analytical chemistry
$^{13}C_6$-rT3	N/A	N/A	Cerilliant Corporation	> 99%	Analytical chemistry
L-thyroxine (T4)	300-30-1	0023662	Cerilliant Corporation	> 98%	Analytical chemistry
$^{13}C_6$-T4	N/A	N/A	Cerilliant Corporation	> 98%	Analytical chemistry

*Denotes chemicals procured through US EPA contract#68HE0D18D0001 with EvoTec Inc. (Branford, CT), who provided dosing solutions solubilized in 95% ethanol.

Supporting Information

Table S4. Mobile phase gradient for targeted analysis of thyroid hormones in plasma on a Sciex 6500+ QTRAP. Both mobile phases contained 0.1% formic acid as an additive.

Time (min)	%A (H_2O)	%B (MeOH)
0.01	70	30
3.89	30	70
4.66	30	70
4.67	10	90
6.22	10	90
6.23	70	30
8.55	70	30
8.56	System Controller Stop	

Table S5. Various instrument conditions for plasma thyroid hormone quantitation on a Sciex 6500+ QTRAP.

Sciex 6500+ Parameter	Setting
Source	ESI
Polarity	Positive
Scan Type	MRM
Source Temperature (°C)	500
Spray Voltage (kV)	5.5
Curtain Gas (psi)	35
Ion Source Gas 1 (psi)	90
Ion Source Gas 2 (psi)	80
Collision Gas	Medium
Detection Window (sec)	80
Scan Time (sec)	0.33

Table S4. Monitored transitions for analysis of thyroid hormones and ^{13}C-labeled internal standards on a Sciex 6500+ QTRAP. All ions were acquired in positive ion mode.

Analyte	Precursor Ion	Fragment Ion	Declustering Potential (DP)	Collision Energy (CE)	Transition Type
T3	651.80	605.7	100	100.0	Quant
	651.80	478.9	100	50.5	Qual
^{13}C-T3	657.80	605.7	100	33.0	IS
rT3	651.75	605.7	100	34.0	Quant
	651.75	508.1	100	35.0	Qual
^{13}C-rT3	657.80	605.7	100	33.0	IS
T4	777.70	731.7	100	40.0	Quant
	777.70	605.1	100	58.3	Qual
^{13}C-T4	783.70	737.7	100	53.0	IS

Quant: Quantifier ion; Qual: Qualifier ion; IS: Internal standard ion.

Table S5. Mobile phase gradient for targeted analysis of HFPO-TeA on a Sciex X500R QTOF/MS. Both mobile phases contained ammonium formate (4 mM) as an additive.

Time (min)	%A (95:5 H_2O/MeOH)	%B (95:5 MeOH/H_2O)
0.0	98	2
1.0	40	60
5.0	0	100
10.0	0	100
10.1	98	2
15	98	2

Table S6. Various instrument conditions for sample analysis on a Sciex X500R QTOF/MS.

Sciex X500R Parameter	MRM Targeted Analysis	Information Dependent Analysis Non-Targeted	Information Independent Analysis Non-Targeted
Source	ESI	ESI	ESI
Polarity	Negative	Negative	Negative
Scan Type	MRM	IDA	SWATH
Ion Source Gas 1 (psi)	30	30	30
Ion Source Gas 2 (psi)	30	35	35
Curtain Gas (psi)	30	30	30
CAD Gas	8	8	8
Source Temperature (°C)	400	400	400
Spray Voltage (V)	-4500	-3750	-3750
TOF MS Scan Range (Da)	100-1000	100-1250	100-1500
TOF MS DP (V)	-95	-50	-50
TOF MS DP Spread (V)	0	0	0
TOF MS CE (V)	-10	-5	-5
TOF MS CE Spread (V)	0	0	0
TOF MS Accumulation Time (sec)	0.25	0.1	0.1
TOF MSMS Scan Range (Da)	N/A	50-1250	50-1250
TOF MSMS Accumulation Time (sec)	0.1	0.05	0.05
Monitored Transition 1 (MT1)	350.97 → 184.9856	N/A	N/A
MT1 TOF MSMS DP (V)	-25	N/A	N/A
MT1 TOF MSMS CE (V)	-45	N/A	N/A
Monitored Transition 2 (MT2)	350.97 → 118.9919	N/A	N/A
MT2 TOF MSMS DP (V)	-25	N/A	N/A
MT2 TOF MSMS CE (V)	-35	N/A	N/A
Monitored Transition 3 (MT3)	812.95 → 768.9514	N/A	N/A
MT3 TOF MSMS DP (V)	-25	N/A	N/A
MT3 TOF MSMS CE (V)	-15	N/A	N/A
TOF MSMS DP (V)	N/A	-40	-40
TOF MSMS DP Spread (V)	0	0	0
TOF MSMS CE (V)	N/A	-30	-30
TOF MSMS CE Spread (V)	N/A	15	15
Maximum Candidate Ions	N/A	15	N/A
Minimum Intensity Threshold (CPS)	N/A	100	N/A
Total Scan Time (sec)	0.612	0.927	0.954
Estimated Cycles	1471	2931	2831

Table S7. Monitored transitions for analysis of HFPO-TeA using PFHxDA as an internal standard on a Sciex X500R QTOF/MS. The ion of m/z 350.97 is the in-source fragment formed from the HFPO-TeA molecular ion of m/z 660.97. All ions were acquired in negative ion mode.

Analyte	Precursor Ion	Fragment Ion	DP	CE	Transition Type
HFPO-TeA	350.97	184.9856	-25	-45	Quant
		118.9919	-25	-35	Qual
PFHxDA	812.95	768.9514	-25	-15	IS

Quant: Quantifier ion; Qual: Qualifier ion; IS: Internal standard ion.

Table S8. Mobile phase gradient for non-targeted analysis on a Sciex X500R QTOF/MS. Ammonium formate (4 mM) was present in both mobile phases as an additive.

Time (min)	%A (95:5 H_2O/MeOH)	%B (95:5 MeOH/H_2O)
0.0	98	2
1.0	90	10
25.0	0	100
30.0	0	100
30.1	98	2
45.0	98	2

Table S9. Mobile phase gradient for targeted analysis of HFPO-TeA on a Waters Xevo-TQS. Both mobile phases contained the additive ammonium acetate (2.5 mM).

Time (min)	%A (95:5 H_2O/ACN)	%B (95:5 ACN/H_2O)
0	95	5
2	95	5
2.45	80	20
2.6	50	50
3.5	42	58
4.25	34	66
4.4	25	75
5.6	20	80
5.9	0	100
7.64	0	100
7.7	80	20
8	80	20
9.5	95	5

Table S10. Various instrument conditions for hepatocyte clearance and protein plasma binding assays on a Waters Xevo-TQS.

Xevo TQ-S Micro Parameter	Setting
Source	UniSpray (US)
Polarity	Positive/Negative
Scan Type	MRM
Capillary Voltage (kV)	1.00
Cone Gas Flow (L/Hr)	0
Desolvation Gas Flow (L/Hr)	650
Source Temperature (°C)	110

Table S11. Monitored transitions for analysis of in vitro analytes using a Waters Xevo-TQS.

Analyte	Precursor Ion	Fragment Ion	Cone (V)	CE	Transition Type	Ion Mode
HFPO-DA	285.08	169.02	2.00	6.0	Quant	Negative
		185.03		12	Qual	
HFPO-TA	494.93	118.89	42.00	40	Quant	Negative
		184.97		8.0	Qual	
HFPO-TeA	350.97	118.90	40.00	36	Quant	Negative
		184.92		10	Qual	
$^{13}C_3$-HFPO-DA	286.97	168.96	12.00	6.0	Quant	Negative
		184.97		16	Qual	
Testosterone	289.20	97.10	33.00	20	Quant	Positive
		109.10		15	Qual	
^{13}C-Testosterone	292.02	100.02	42.00	22	Quant	Positive
		111.98		24	Qual	
Phenacetin	180.12	92.89	32.00	28	Quant	Positive
		110.00		22	Qual	
Propranolol	260.18	55.92	22.00	30	Quant	Positive
		116.00		18	Qual	

Quant: Quantifier ion; Qual: Qualifier ion

Table S12. Individual body weights, absolute liver weights, and relative liver weights for all rats after 5 days of exposure.

Rat ID	Dose Level (mg/kg/day)	Sex	Beginning Body Weight (g)	Terminal Body Weight (g)	Body Weight Change (g)	Avg. ± St. Dev. Body Weight Change (g)	Liver Weight (g)	Relative Liver Weight (g%)	Average ± St. Dev. Relative Liver Weight (g%)
R145	0	M	228.1	323.3	35.2		15.0154	4.644	
R146	0	M	284.3	315.0	30.7	31.3 ± 3.3	15.2084	4.828	4.577 ± 0.236
R147	0	M	283.2	315.4	32.2		14.4240	4.573	
R148	0	M	286.4	313.6	27.2		13.3673	4.263	
R149	0.3	M	285.6	313.8	28.2		13.9494	4.445	
R150	0.3	M	290.6	330.7	40.1	37.2 ± 9.0	16.3796	4.953	4.702 ± 0.220
R151	0.3	M	286.4	334.9	48.5		15.4580	4.616	
R152	0.3	M	286.0	318.0	32.0		15.2495	4.795	
R153	0.9	M	274.5	320.5	46.0		16.7977	5.241	
R154	0.9	M	277.8	316.1	38.3	39.6 ± 4.3	16.8117	5.318	5.291 ± 0.201
R155	0.9	M	286.5	323.9	37.4		16.3850	5.059	
R156	0.9	M	298.3	335.0	36.7		18.5738	5.544	
R157	2.3	M	286.1	323.6	37.5		17.9851	5.558	
R158	2.3	M	279.6	325.4	45.8	39.0 ± 4.8	19.2395	5.913	5.916 ± 0.297
R159	2.3	M	294.6	332.8	38.2		20.9175	6.285	
R160	2.3	M	283.3	317.9	34.6		18.7875	5.910	
R161	6.3	M	281.4	308.9	27.5		19.3187	6.254	
R162	6.3	M	301.7	344.9	43.2	31.8 ± 8.1	25.7576	7.468	6.983 ± 0.527
R163	6.3	M	290.0	321.7	31.7		22.4062	6.965	
R164	6.3	M	283.7	308.4	24.7		22.3377	7.243	
R165	17	M	286.9	250.6	-36.3		13.4969	5.386	
R166	17	M	298.7	248.0	-50.7	-51.5 ± 10.9	13.8697	5.593	5.600 ± 0.223
R167	17	M	286.5	226.8	-59.7		12.4995	5.511	
R168	17	M	281.1	221.9	-59.2		13.1122	5.909	
R181	0	F	231.4	239.4	8.0		10.6198	4.436	
R182	0	F	226.1	232.1	6.0	3.7 ± 5.7	9.6526	4.159	4.190 ± 0.189
R183	0	F	220.1	225.6	5.5		8.9720	3.977	
R184	0	F	225.1	220.5	-4.6		9.2394	4.190	
R185	0.3	F	219.6	230.9	11.3		10.2929	4.458	
R186	0.3	F	217.4	225.9	8.5	6.5 ± 4.4	10.0087	4.431	4.488 ± 0.062
R187	0.3	F	228.7	229.7	1.0		10.5040	4.573	
R188	0.3	F	221.8	226.9	5.1		10.1866	4.489	
R189	0.9	F	229.1	237.7	8.6		12.6621	5.327	
R190	0.9	F	208.7	227.1	18.4	12.2 ± 4.3	11.3910	5.016	5.086 ± 0.224
R191	0.9	F	219.8	230.2	10.4		11.9531	5.192	
R192	0.9	F	228.8	240.1	11.3		11.5466	4.809	
R193	2.3	F	221.3	229.6	8.3		12.3341	5.372	
R194	2.3	F	224.4	245.9	21.5	12.3 ± 6.2	12.8755	5.236	5.296 ± 0.073
R195	2.3	F	214.7	225.6	10.9		12.0585	5.345	
R196	2.3	F	229.5	238.0	8.5		12.4504	5.231	
R197	6.3	F	225.6	213.8	-11.8		10.2774	4.807	
R198	6.3	F	210.3	189.0	-21.3	-17.8 ± 14.3	9.4969	5.025	5.329 ± 0.634
R199	6.3	F	210.1	207.9	-2.2		12.9764	6.242	
R200	6.3	F	215.5	179.8	-35.7		9.4275	5.243	
R201	17	F	224.5	161.8	-62.7		9.7114	6.002	
R202	17	F	227.0	174.9	-52.1	-55.2 ± 5.7	9.8972	5.659	5.796 ± 0.216
R203	17	F	214.4	164.8	-49.6		9.1738	5.567	
R204	17	F	222.9	166.6	-56.3		9.9260	5.958	

Table S13. Individual concentrations for plasma T3, rT3, and T4 in all rats after 5 days of exposure to HFPO-TeA. < LOQ = sample concentration was below the LOQ (rT3 =0.005 ng/mL). N/A = Calculation not completed due the majority of samples being below the LOQ.

Rat ID	Dose Level (mg/kg/day)	Sex	T3 Conc. (ng/mL)	Avg. ± St. Dev. T3 Conc. (ng/mL)	rT3 Conc. (ng/mL)	Avg. ± St. Dev. rT3 Conc. (ng/mL)	T4 Conc. (ng/mL)	Avg. ± St. Dev. T4 Conc. (ng/mL)
R145	0	M	0.644	0.737 ± 0.066	< LOQ	0.0510 ± N/A	35.3	39.8 ± 3.6
R146	0	M	0.736		0.0510		41.6	
R147	0	M	0.775		< LOQ		38.5	
R148	0	M	0.793		< LOQ		43.6	
R149	0.3	M	0.920	0.811 ± 0.111	0.127	0.127 ± N/A	50.1	37.6 ± 10.4
R150	0.3	M	0.695		< LOQ		36.3	
R151	0.3	M	0.891		< LOQ		24.7	
R152	0.3	M	0.739		< LOQ		39.4	
R153	0.9	M	0.829	0.723 ± 0.105	< LOQ	N/A	37.5	38.8 ± 5.2
R154	0.9	M	0.783		< LOQ		37.0	
R155	0.9	M	0.687		< LOQ		46.3	
R156	0.9	M	0.594		< LOQ		34.4	
R157	2.3	M	0.633	0.678 ± 0.101	0.188	0.188 ± N/A	36.0	35.8 ± 10.9
R158	2.3	M	0.764		< LOQ		37.6	
R159	2.3	M	0.556		< LOQ		48.1	
R160	2.3	M	0.759		< LOQ		21.5	
R161	6.3	M	0.680	0.630 ± 0.060	0.061	0.0847 ± 0.0257	30.9	32.9 ± 6.9
R162	6.3	M	0.627		0.081		26.7	
R163	6.3	M	0.667		0.112		31.3	
R164	6.3	M	0.547		< LOQ		42.7	
R165	17	M	0.334	0.423 ± 0.078	< LOQ	0.0630 ± N/A	8.34	9.82 ± 2.94
R166	17	M	0.518		0.0630		14.0	
R167	17	M	0.444		< LOQ		9.60	
R168	17	M	0.394		< LOQ		7.34	
R181	0	F	0.717	0.870 ± 0.200	< LOQ	0.153 ± N/A	35.9	29.7 ± 4.3
R182	0	F	0.689		< LOQ		26.0	
R183	0	F	0.973		< LOQ		28.5	
R184	0	F	1.10		0.153		28.2	
R185	0.3	F	1.00	0.844 ± 0.106	< LOQ	0.143 ± N/A	30.9	31.3 ± 3.5
R186	0.3	F	0.775		< LOQ		36.4	
R187	0.3	F	0.784		< LOQ		29.1	
R188	0.3	F	0.817		0.143		28.9	
R189	0.9	F	0.974	0.799 ± 0.167	< LOQ	N/A	30.2	26.8 ± 4.2
R190	0.9	F	0.862		< LOQ		29.2	
R191	0.9	F	0.782		< LOQ		20.9	
R192	0.9	F	0.577		< LOQ		26.9	
R193	2.3	F	0.783	0.781 ± 0.024	0.0960	0.105 ± 0.012	35.6	34.9 ± 5.8
R194	2.3	F	0.750		< LOQ		38.4	
R195	2.3	F	0.782		0.113		26.5	
R196	2.3	F	0.809		< LOQ		38.9	
R197	6.3	F	0.729	0.563 ± 0.162	< LOQ	0.0860 ± N/A	26.4	19.9 ± 7.1
R198	6.3	F	0.399		< LOQ		16.8	
R199	6.3	F	0.672		0.0860		24.9	
R200	6.3	F	0.451		< LOQ		11.3	
R201	17	F	0.806	0.585 ± 0.160	0.0480	0.0510 ± 0.0042	21.9	15.9 ± 5.9
R202	17	F	0.548		< LOQ		16.2	
R203	17	F	0.425		< LOQ		7.77	
R204	17	F	0.562		0.0540		17.9	

Table S14. Individual HFPO-TeA plasma and plasma extract concentrations for all rats after 2 hours of exposure. < LOQ = sample concentration was below the LOQ (20 ng/mL or 0.0302 μM) N/A = not applicable due to data being below the LOQ.

Rat ID	Dose Level (mg/kg/day)	Sex	Plasma Extract Conc. (ng/mL)	Dilution Factor	Plasma Conc. (μg/mL)	Plasma Conc. (μM)	Avg. ± St. Dev. Plasma Conc. (μM)
R145	0	M	ND	25	< LOQ	< LOQ	N/A
R146	0	M	ND	25	< LOQ	< LOQ	
R147	0	M	ND	25	< LOQ	< LOQ	
R148	0	M	ND	25	< LOQ	< LOQ	
R149	0.3	M	4.66	25	0.117	0.176	0.150 ± 0.020
R150	0.3	M	3.76	25	0.094	0.142	
R151	0.3	M	3.42	25	0.086	0.129	
R152	0.3	M	4.05	25	0.101	0.153	
R153	0.9	M	8.62	50	0.431	0.651	0.573 ± 0.113
R154	0.9	M	5.81	50	0.291	0.439	
R155	0.9	M	8.99	50	0.450	0.679	
R156	0.9	M	6.90	50	0.345	0.521	
R157	2.3	M	19.1	400	7.65	11.6	13.1 ± 2.8
R158	2.3	M	28.6	400	11.4	17.3	
R159	2.3	M	20.1	400	8.06	12.2	
R160	2.3	M	18.6	400	7.45	11.3	
R161	6.3	M	59.3	400	23.7	35.8	34.0 ± 5.5
R162	6.3	M	61.0	400	24.4	36.8	
R163	6.3	M	42.7	400	17.1	25.8	
R164	6.3	M	62.4	400	24.9	37.7	
R165	17	M	152	400	60.6	91.6	126 ± 62
R166	17	M	139	400	55.8	84.3	
R167	17	M	183	400	73.0	110	
R168	17	M	361	400	114.0	218	
R181	0	F	ND	25	< LOQ	< LOQ	N/A
R182	0	F	ND	25	< LOQ	< LOQ	
R183	0	F	ND	25	< LOQ	< LOQ	
R184	0	F	ND	25	< LOQ	< LOQ	
R185	0.3	F	3.28	25	0.0820	0.124	0.137 ± 0.033
R186	0.3	F	4.94	25	0.124	0.187	
R187	0.3	F	3.11	25	0.0778	0.117	
R188	0.3	F	3.14	25	0.0785	0.119	
R189	0.9	F	5.23	100	0.523	0.790	0.620 ± 0.133
R190	0.9	F	6.18	50	0.309	0.467	
R191	0.9	F	8.36	50	0.418	0.631	
R192	0.9	F	7.84	50	0.392	0.592	
R193	2.3	F	17.2	400	6.87	10.4	15.1 ± 5.3
R194	2.3	F	19.4	400	7.78	11.7	
R195	2.3	F	26.9	400	10.8	16.3	
R196	2.3	F	36.5	400	14.6	22.1	
R197	6.3	F	49.8	400	19.5	29.5	53.2 ± 16.5
R198	6.3	F	110	400	43.9	66.4	
R199	6.3	F	103	400	41.1	62.0	
R200	6.3	F	90.9	400	36.3	54.9	
R201	17	F	494	400	197	298	224 ± 76
R202	17	F	460	400	184	278	
R203	17	F	235	400	94.0	142	
R204	17	F	295	400	118	179	

Table S15. Individual HFPO-TeA plasma and plasma extract concentrations for all rats after 5 days of exposure. < LOQ = sample concentration was below the LOQ (20 ng/mL or 0.0302 μM) N/A = not applicable due to data being below the LOQ.

Rat ID	Dose Level (mg/kg/day)	Sex	Plasma Extract Conc. (ng/mL)	Dilution Factor	Plasma Conc. (μg/mL)	Plasma Conc. (μM)	Avg. ± St. Dev. Plasma Conc. (μM)
R145	0	M	ND	25	< LOQ	< LOQ	N/A
R146	0	M	ND	25	< LOQ	< LOQ	
R147	0	M	ND	25	< LOQ	< LOQ	
R148	0	M	ND	25	< LOQ	< LOQ	
R149	0.3	M	22.3	25	0.558	0.843	0.827 ± 0.071
R150	0.3	M	23.4	25	0.586	0.885	
R151	0.3	M	22.7	25	0.567	0.856	
R152	0.3	M	19.2	25	0.479	0.723	
R153	0.9	M	2.34	800	1.87	2.83	2.73 ± 0.45
R154	0.9	M	1.81	800	1.45	2.19	
R155	0.9	M	2.70	800	2.16	3.26	
R156	0.9	M	2.19	800	1.75	2.65	
R157	2.3	M	7.10	800	5.68	8.58	6.64± 1.73
R158	2.3	M	4.19	800	3.35	5.06	
R159	2.3	M	4.38	800	3.50	5.29	
R160	2.3	M	6.30	800	5.04	7.61	
R161	6.3	M	19.3	800	15.5	23.3	24.5 ± 2.3
R162	6.3	M	18.9	800	15.1	22.9	
R163	6.3	M	19.7	800	5.7	23.8	
R164	6.3	M	23.1	800	18.5	28.0	
R165	17	M	43.3	1600	65.4	98.9	168 ± 53
R166	17	M	69.6	1600	109	165	
R167	17	M	90.6	1600	150	227	
R168	17	M	74.6	1600	118	179	
R181	0	F	ND	25	< LOQ	< LOQ	N/A
R182	0	F	ND	25	< LOQ	< LOQ	
R183	0	F	ND	25	< LOQ	< LOQ	
R184	0	F	ND	25	< LOQ	< LOQ	
R185	0.3	F	22.0	25	0.550	0.831	0.854 ± 0.086
R186	0.3	F	19.7	25	0.492	0.743	
R187	0.3	F	24.9	25	0.622	0.939	
R188	0.3	F	23.9	25	0.596	0.901	
R189	0.9	F	2.34	800	1.87	2.83	3.62 ± 0.91
R190	0.9	F	2.44	800	1.95	2.95	
R191	0.9	F	3.95	800	3.16	4.77	
R192	0.9	F	3.25	800	2.60	3.93	
R193	2.3	F	7.13	800	5.70	8.62	8.92 ± 2.36
R194	2.3	F	5.40	800	4.32	6.53	
R195	2.3	F	6.92	800	5.54	8.36	
R196	2.3	F	10.1	800	8.06	12.2	
R197	6.3	F	42.4	800	33.9	51.2	52.6 ± 14.3
R198	6.3	F	39.5	800	31.6	47.8	
R199	6.3	F	32.0	800	25.6	38.7	
R200	6.3	F	60.1	800	48.0	72.6	
R201	17	F	116	1600	200	302	278 ± 28
R202	17	F	94.6	1600	160	241	
R203	17	F	102	1600	181	273	
R204	17	F	123	1600	196	296	

Table S16. Individual HFPO-TeA liver (wet weight; ww) and liver extract concentrations for all rats after 5 days of exposure. < LOQ = sample concentration was below the LOQ (0.99 ng/mg ww or 1.58 μM) N/A = not applicable due to data being below the LOQ.

Rat ID	Dose Level (mg/kg/day)	Sex	Liver Extract Conc. (ng/mL)	Dilution Factor	Liver Conc. (ng/mg)	Liver Conc. (μM)	Avg. ± St. Dev. Liver Conc. (μM)
R145	0	M	ND	1	< LOQ	< LOQ	N/A
R146	0	M	ND	1	< LOQ	< LOQ	
R147	0	M	ND	1	< LOQ	< LOQ	
R148	0	M	ND	1	< LOQ	< LOQ	
R149	0.3	M	35.5	1	1.85	2950	6070 ± 3410
R150	0.3	M	138	1	6.84	10,900	
R151	0.3	M	71.6	1	3.28	5240	
R152	0.3	M	57.0	1	3.23	5160	
R153	0.9	M	60.4	2	3.10	4950	9060 ± 3170
R154	0.9	M	109	2	5.32	8500	
R155	0.9	M	125	2	6.52	10,400	
R156	0.9	M	159	2	7.76	12,400	
R157	2.3	M	82.5	2	4.09	6530	10,300 ± 7530
R158	2.3	M	98.5	2	4.65	7430	
R159	2.3	M	70.3	2	3.64	5810	
R160	2.3	M	282	5	13.5	21,600	
R161	6.3	M	1130	20	61.6	98,400	82,700 ± 10,500
R162	6.3	M	1100	20	48.9	78,100	
R163	6.3	M	937	1	48.1	76,800	
R164	6.3	M	1050	1	48.5	77,400	
R165	17	M	2340	30	109	174,000	210,000 ± 84,900
R166	17	M	1360	30	80.8	129,000	
R167	17	M	2200	3	132	211,000	
R168	17	M	4030	3	205	327,000	
R181	0	F	ND	1	< LOQ	< LOQ	N/A
R182	0	F	ND	1	< LOQ	< LOQ	
R183	0	F	ND	1	< LOQ	< LOQ	
R184	0	F	ND	1	< LOQ	< LOQ	
R185	0.3	F	83.2	1	4.09	6530	6380 ± 3140
R186	0.3	F	130	1	6.67	10,700	
R187	0.3	F	69.7	1	3.13	5060	
R188	0.3	F	33.3	1	2.06	3290	
R189	0.9	F	171	1	9.24	14,800	14,000 ± 4280
R190	0.9	F	123	2	6.65	10,600	
R191	0.9	F	121	1	6.85	10,900	
R192	0.9	F	223	1	12.4	19,800	
R193	2.3	F	427	5	21.5	34,300	16,500 ± 12,600
R194	2.3	F	81.6	2	3.65	5830	
R195	2.3	F	202	5	9.93	15,900	
R196	2.3	F	111	5	6.28	10,000	
R197	6.3	F	738	20	42.2	67,400	86,700 ± 31,700
R198	6.3	F	1200	1	69.2	110,000	
R199	6.3	F	695	20	32.8	52,400	
R200	6.3	F	1376	20	73.1	117,000	
R201	17	F	3600	3	174	278,000	250,000 ± 39,300
R202	17	F	2780	3	160	255,000	
R203	17	F	2429	30	121	193,000	
R204	17	F	3790	3	172	275,000	

Supporting Information

References

1. Probst, R.J.; Lim, J.M.; Bird, D.N.; Pole, G.L.; Sato, A.K.; Claybaugh, J.R. Gender Differences in the Blood Volume of Conscious Sprague-Dawley Rats. *Journal of the American Association for Laboratory Animal Science* **2006**, *45*, 49-52.
2. Trudnowski, R.J.; Rodolfo, R.C. Specific Gravity of Blood and Plasma at 4 and 37 °C. *Clinical Chemistry* **1974**, *20*, 615-616, doi:10.1093/clinchem/20.5.615.
3. Kim, S.-J.; Heo, S.-H.; Lee, D.-S.; Hwang, I.G.; Lee, Y.-B.; Cho, H.-Y. Gender differences in pharmacokinetics and tissue distribution of 3 perfluoroalkyl and polyfluoroalkyl substances in rats. *Food and Chemical Toxicology* **2019**, *97*, 243-255, doi:10.1016/j.fct.2016.09.017.
4. Yi, S.; Yang, D.; Zhu, L.; Mabury, S.A. Significant Reductive Transformation of 6:2 Chlorinated Polyfluorooctane Ether Sulfonate to Form Hydrogen-Substituted Polyfluorooctane Ether Sulfonate and Their Toxicokinetics in Male Sprague-Dawley Rats. *Environmental Science & Technology* **2022**, *56*, 6123-6132, doi:10.1021/acs.est.1c00616.
5. Jia, Y.; Zhu, Y.; Xu, D.; Feng, X.; Yu, X.; Shan, G.; Zhu, L. Insights into the Competitive Mechanisms of Per- and Polyfluoroalkyl Substances Partition in Liver and Blood. *Environmental Science & Technology* **2022**, *56*, 6192-6200, doi:10.1021/acs.est.1c08493.
6. Lambert, J.-P.; Ivosev, G.; Couzens, A.L.; Larsen, B.; Taipale, M.; Lin, Z.-Y.; Zhong, Q.; Lindquist, S.; Vidal, M.; Aebersold, R.; et al. Mapping differential interactomes by affinity purification coupled with data independent mass spectrometry acquisition. *Nature Methods* **2013**, *10*, 1239-1245, doi:10.1038/nmeth.2702.
7. Yao, J.; Sheng, N.; Guo, Y.; Yeung, L.W.Y.; Dai, J.; Pan, Y. Nontargeted Identification and Temporal Trends of Per- and Polyfluoroalkyl Substances in a Fluorochemical Industrial Zone and Adjacent Taihu Lake. *Environmental Science & Technology* **2022**, *56*, 7986-7996, doi:10.1021/acs.est.2c00891.
8. Wetmore, B.A. Quantitative *in vitro*--to-*in vivo* extrapolation in a high-throughput environment. *Toxicology* **2015**, *332*, 94-101, doi:10.1016/j.tox.2014.05.012.
9. Wilkinson, G.R.; Shand, D.G. A physiological approach to hepatic drug clearance. *Clinical Pharmacology & Therapeutics* **1975**, *18*, 377-390, doi:10.1002/cpt1975184377.
